# Synthesis, Anti-Cancer and Anti-Migratory Evaluation of 3,6-Dibromocarbazole and 5-Bromoindole Derivatives

**DOI:** 10.3390/molecules24152686

**Published:** 2019-07-24

**Authors:** Krystal M. Butler-Fernández, Zulma Ramos, Adela M. Francis-Malavé, Joseph Bloom, Suranganie Dharmawardhane, Eliud Hernández

**Affiliations:** 1 Department of Pharmaceutical Sciences, University of Puerto Rico, School of Pharmacy, San Juan 00936, Puerto Rico; 2 Department of Biology, College of Natural Sciences, University of Puerto Rico, San Juan 00931, Puerto Rico; 3 Department of Biochemistry, University of Puerto Rico, School of Medicine, San Juan 00936, Puerto Rico

**Keywords:** 3,6-dibromocarbazole, 5-bromoindole, carbazole, actin, breast cancer, migration

## Abstract

In this study, a new series of *N*-alkyl-3,6-dibromocarbazole and *N*-alkyl-5-bromoindole derivatives have been synthesized and evaluated in vitro as anti-cancer and anti-migration agents. Cytotoxic and anti-migratory effects of these compounds were evaluated in MCF-7 and MDA-MB-231 breast cancer cell lines and an insight on the structure-activity relationship was developed. Preliminary investigations of their anti-cancer activity demonstrated that several compounds have moderate antiproliferative effects on cancer cell lines with GI_50_ values in the range of 4.7–32.2 µM. Moreover, carbazole derivatives **10**, **14**, **15**, **23**, and **24** inhibit migration activity of metastatic cell line MDA-MB-231 in the range of 18–20%. The effect of compounds **10**, **14**, and **15** in extension of invadopodia and filopodia was evaluated by fluorescence microscopy and results demonstrated a reduction in actin-based cell extensions by compounds **10** and **15**.

## 1. Introduction

In women, breast cancer is the leading cause of death, mainly due to metastasis [1]. If breast cancer is detected and treated prior to metastasis, the patient has a higher probability of being cured of their disease. Cancer cell invasion involves cell migration through the extracellular matrix (ECM) and the accompanying degradation of the ECM [2]. Several proteins play a key role in this process, by the extension of structures known as invadopodia. Invadopodia are actin-rich protrusive structures with associated matrix degradation activity and are believed to be important for tumor cells to penetrate the basement membrane of epithelia and blood vessels [3]. In cell migration, the reorganization of the actin cytoskeleton produces the force necessary for cell migration [4]. The Rho GTPases, Rac, and Cdc42 are key molecular switches activated by a myriad of cell surface receptors to promote breast cancer cell migration/invasion, proliferation, and survival [5]. Unlike Ras, Rac and Cdc42 are not mutated in breast cancer, but activated via the deregulation of expression and/or activity of their upstream regulators, guanine nucleotide exchange factors (GEFs) [6]. The WASP family proteins are key regulators of the actin cytoskeleton and cell migration through induction of membrane protrusions at the leading edge [7]. In cancer cells, when N-WASP interacts and activates the Arp2/3 complex, it catalyzes actin polymerization and assembly into filopodia and invadopodia [7,8]. To initiate this process, the Rho GTPase Cdc42, in its GTP-activated form, binds and activates N-WASP by inducing a conformational change that liberates the autoinhibited structure, thereby interacting with Arp2/3 complex, and regulating the protrusive formation in membrane structures promoting extracellular matrix (ECM) degradation [8]. Therefore, inhibition of these processes decreases cell motility and invasion, and may greatly improve the potential therapeutic applications of such inhibitors against cancer metastasis.

**Figure 1:**
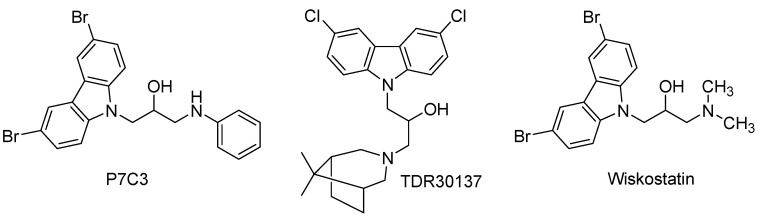
Structure of representative *N*-alkyl-3,6-dihalogencarbazole derivatives.

Natural and synthetic carbazole derivatives comprise a wide variety of biologically active agents with diverse pharmacological activities, including antitumoral, antioxidant, anti-inflammatory, antibacterial, anticonvulsant, antipsychotic, antidiabetic, and larvicidal properties [9,10,11,12,13,14,15,16,17,18,19,20,21,22,23,24,25,26,27,28]. Carbazoles are tricyclic aromatic compounds with a benzene ring fused to the 2,3-positions of an indole ring [29]. The antitumor properties of carbazole derivatives have been correlated to their polycyclic, planar aromatic structure, and large π-conjugated backbone that noncovalently bind with DNA base pairs, hydrophobic pockets, and forms electrostatic interactions to intercalate into DNA [9,30]. In particular, among a wide variety of carbazoles, a series of *N*-alkyl-3,6-disubstituted carbazole derivatives has been discovered and evaluated for their potential as neuroprotective agents [31], antimalarial [32], antitumoral [33,34], anti-apoptotic [35], and antibacterial activities [36]. Selected examples of bioactive *N*-alkyl-3,6-disubstituted carbazole derivatives are represented in Figure 1.

The compound P7C3 (Figure 1) was discovered from a library of 200,000 drug-like molecules, and showed proneurogenic and neuroprotective properties, stabilized mitochondrial membrane potential, and inhibits neuronal apoptosis [37]. Several derivatives of P7C3 have been synthesized with modifications at the linker chain. Replacement of the hydroxyl group with a fluorine atom, and an additional methylene group between the hydroxyl group and the aniline, increases activity [37]. The aromatic ring was replaced with heteroaromatic groups, but activity was found to be less effective. The commercially available TDR30137 (Figure 1) was discovered and characterized as an inhibitor of P. *falciparum* K1 (Pf-K1) in human red blood cells with an IC_50_ of 57 nM [32]. However, TDR30137 was not active in in vivo studies with the *Plasmodium berghei* mouse model. In structure-activity relationship (SAR) studies, the importance of 3,6-halogen substitution, hydroxyl group, and the tertiary amine correlated with improved activity on Pf-K1 strains [32]. A carbazole derivative named Wiskostatin (Figure 1) was identified to bind within a pocket in the GBD regulatory module that maintains N-WASP in an inactive, autoinhibited conformation [38]. In a pyrene-actin polymerization assay, using purified proteins, it was demonstrated that Wiskostatin inhibited full-length N-WASP activation of the Arp2/3 complex at IC_50_ = 10 μM [38]. The specific binding site of Wiskostatin was determined to be within the GBD of the autoinhibited conformation of N-WASP after performing the experiment with activated Cdc42-GTP. Unfortunately, a recent report described that Wiskostatin inhibited other cellular functions that are not believed to be N-WASP dependent [39]. These studies revealed that Wiskostatin caused an irreversible decrease in cellular ATP levels, and that it does not function as a selective inhibitor of N-WASP dependent functions in intact cells, and caused an overall change in the energy status of cells; thus, inhibiting normal transport processes [39]. Herein, we designed and synthesized a new series of *N*-alkyl-3,6-dibromocarbazole and 5-bromoindole derivatives, tested for their antiproliferative and antimigratory activities in MCF-7 and MDA-MB-231 breast cancer cell lines, and analyzed the effect of the most active migration inhibitor on actin dynamics and actin cytoskeleton rearrangement.

## 2. Results and Discussion

The aim of this study is to design and synthesize a new series of *N*-alkyl-3,6-dibromocarbazole and *N*-alkyl-5-bromoindole derivatives and analyze their cytotoxic effect and potential to inhibit actin cytoskeleton rearrangement and cancer cell migration. The structural elements of Wiskostatin and derivatives identified as pharmacophoric unit are a 3,6-dihalogen carbazole and a dialkylamino-2-propanol chain. Our strategy was to design and synthesize a new series of compounds with a 3,6-dibromocarbazole or 5-bromoindole ring connected, via a three-carbon atom aliphatic chain, to an amide group. The influence of different N-alkyl or aromatic substituents at the amide group was examined.

**Figure 2:**
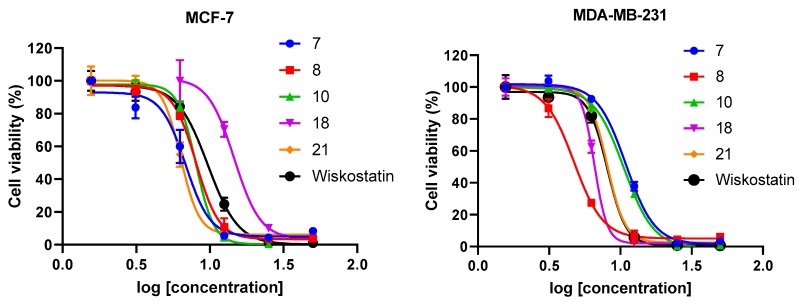
Log-dose response curve for compounds **7**, **8**, **10**, **18**, **21**, and Wiskostatin on MCF-7 and MDA-MB-231 breast cancer cell lines. Each data point represents the mean of three replicates and error bars represent ± SD. Each GI_50_ value was calculated based on sigmoidal curve fitting to the respective data set.

**Figure 3:**
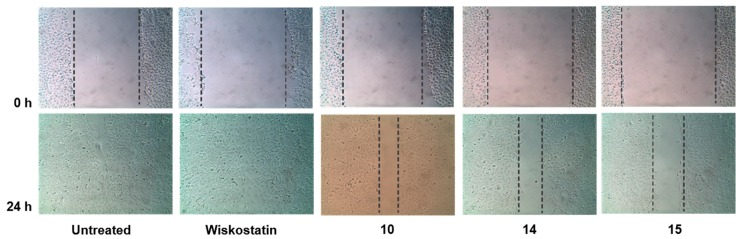
Inhibitory effect of compounds **10**, **14**, **15**, and Wiskostatin on MDA-MB-231 cells migration detected by wound-healing assay. MDA-MB-231 cells were treated with vehicle or with compounds **10**, **14**, **15**, and Wiskostatin. The photomicrographs were obtained at 0 and 24 h. Percent relative migration values are the average of three independent experiments. Dotted lines show the area occupied by the initial scraping for 0 h, and the wound edge for 24 h.

**Figure 4:**
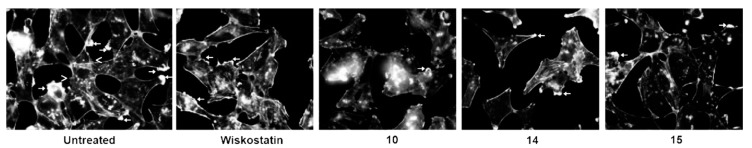
Effect of Wiskostatin and compounds **10**, **14**, and **15** on actin cytoskeleton of metastatic cancer cell MDA-MB-231. MDA-MB-231 metastatic breast cancer cells were treated with vehicle or Wiskostatin (2 µM) and compounds **10**, **14**, or **15** at 10 µM for 24 h to determine changes in actin cytoskeletal structures. Cells were fixed, permeabilized, and stained with rhodamine phalloidin to visualize F-actin. Arrows, lamellipodia; arrowheads, filopodia.

We screened all compounds to determine their cytotoxic effect against MCF-7 and MDA-MB-231 breast cancer cells using the Sulforhodamine B (SRB) assay [40] (Figure 2). In addition, anti-migratory activity was determined using the wound healing assay (scratch method) [41] on the metastatic MDA-MB-231 cancer cells. In this assay, the relative migration of MDA-MB-231 breast cancer cells in the presence of carbazole or indole derivatives at a concentration of 10 µM (or at concentrations that do not affect cell viability) was compared to the migration in the presence of vehicle (0.02% DMSO). Representative photomicrographs of the migration inhibition of compounds **10**, **14**, **15**, and Wiskostatin are represented in Figure 3. Results show that in the vehicle-treated control experiment, wound healing is progressing considerably, and after 24 h, the wound is completely healed. When cells are incubated with compounds **10**, **14**, and **15** after 24 h, the wound healing is inhibited. However, Wiskostatin did not elicit an inhibitory effect on wound healing when incubated with MDA-MB-231 cells after 24 h at a concentration of 2 µM. Actin and Arp2/3 regulation by active WASP induce de novo actin polymerization and assembly to generate the F-actin structures filopodia and invadopodia used for cell migration [42]. Therefore, to investigate the effect of compounds **10**, **14**, **15**, and Wiskostatin on actin dynamics, we performed immunofluorescence microscopy to detect polymerized actin on MDA-MB-231 cancer cells (Figure 4). The structure and biological activities of new compounds are summarized in Table 1 and Table 2.

### 2.1. N-Alkyl-3,6-Dibromocarbazole Derivatives

**Scheme 1:**
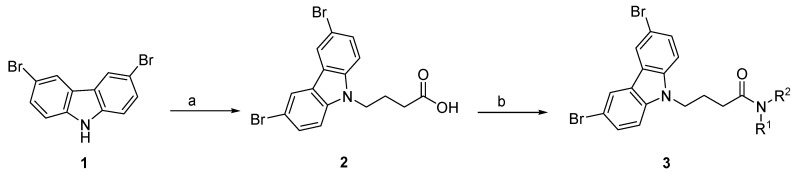
General synthetic procedure of 3,6-dibromocarbazole-4-butyramide derivatives **3**. Reagents and conditions: (**a**) (i) Ethyl 4-bromobutyrate, K_2_CO_3_, DMF, 80 °C, 2h; (ii) KOH, DMF/water, 80 °C, 2-6 h, 86%; (**b**) HOBt, EDAC, CH_2_Cl_2_, Et_3_N, rt, amine: R-NH_2_ or HNR^1^R^2^, 2-8 h.

The synthetic method to construct the 3,6-dibromocarbazole derivatives library is described in Scheme 1 (see the Supplementary Materials for representative ^1^H and ^13^C NMR spectral data). The *N*-alkyl-3,6-dibromocarbazole derivatives were generated in a three-step synthesis using 3,6-dibromocarbazole **1** as origin of the carbazole derivatives core. Compound **1** was reacted with ethyl 4-bromobutyrate to introduce the aliphatic side chain by nucleophilic substitution (Scheme 1), followed by hydrolysis to afford the corresponding 3,6-diromocarbazole-4-butyric acid **2**. For the generation of 3,6-dibromocarbazole-4-butyramide derivatives **3**, compound **2** was therefore used as starting material, which reacted with different amines via an amide coupling reaction using N-(3-Dimethylaminopropyl)-N’-ethylcarbonate (EDAC) with Hydroxybenzotriazole (HOBt) as an additive dissolved in methylene chloride (CH_2_Cl_2_).

The in vitro anti-proliferative and anti-migratory activities of compounds **4**–**27**, and Wiskostatin are represented in Table 1. From the twenty-four compound derivatives of 3,6-dibromocarbazole, it can be observed that in the MCF-7 (ER+) cancer cell line, compounds **6–10**, **14**, **16–21**, and **32** showed good to moderate antiproliferative activity with a GI_50_ in the range of 6.8–32.2 µM. In the MDA-MB-231 cell line, compounds **6–10**, **14**, **16**, **18**, **20–21**, and **23** inhibited cell proliferation with a GI_50_ in the range of 4.7–23 µM. The remaining compounds in that series had a GI_50_ above 50 µM in both breast cancer cell lines. Compound **8** with a 2-piperazinyl ethyl butyramide chain showed very good anticancer activity against both cancer cell lines MCF-7 and MDA-MB-231 with GI_50_ values of 8 and 4.7 µM, respectively. Also, compound **18** with a piperazinyl amide showed significant anticancer activity against both cancer cell lines with GI_50_ values of 7.5 and 6.7 µM, respectively. Similarly, compound **21**, another piperazinyl amide derivative, showed very good in vitro anticancer activity against both cancer cell lines with GI_50_ values of 6.5 and 8 µM, respectively. Shortening the aliphatic chain between the N-atom of the amide group and the morpholine from C3 (**9**), over C2 (**4**), to C0 (**12**) led to a complete loss of antiproliferative activity on both cancer cell lines. On the other hand, introduction of an aromatic ring or aromatic heterocycle (**22–27**, Table 1) in the amide group led to lack of antiproliferative activity with GI_50_ above 50 µM on both cancer cell lines. Thus, in general, compounds with a piperazinyl butyramide group attached to the N-atom of the carbazole appear to be more cytotoxic than compounds with other butyramide group in this series of compounds. In addition, three compounds—**8**, **18**, and **21**—were found to be more cytotoxic against both cancer cell lines MCF-7 and MDA-MB-231 than Wiskostatin, which in this assay showed GI_50_ values of 9.7 and 8.3 µM, respectively (Table 1, Figure 2).

To further assess the anti-migratory activity of carbazole derivatives in vitro, we examined its inhibitory effects on the migration of the metastatic breast cancer cell line MDA-MB-231 using the wound-healing assay at concentrations that do not affect cell viability. We chose the MDA-MB-231 breast cancer cells over MCF-7 due to its enhanced metastatic and migratory properties, with concomitant Rac and Cdc42 expression, compared to the non-metastatic and poorly migrating MCF-7 cells. The relative migration of treated cells with 3,6-dibromocarbazole derivatives compared with control (MDA-MB-231 cells) are summarized in Table 1.

Among the twenty-four compounds tested for their anti-migratory effect, five compounds (**10**, **14**, **15**, **23**, **24**) inhibited migration in the range of 10–20%. While compounds **7**, **8**, **18**, and **21** were among the most cytotoxic compounds tested against MDA-MB-231 and MCF-7 cancer cell lines, they did not show significant anti-migratory effect. In contrast, compounds **14–16** and **24** inhibited migration in the range of 18–20%, compared to Wiskostatin that inhibit 5% of migration at 2 µM. Compound **14**, a carbazole derivative with a piperidine-4-carbonitrile amide group that showed moderate anti-proliferative activity on both MCF-7 and MDA-MB-231 cell lines, inhibit 19% of migration. Both compounds **15** and **16**, with phenyl- and acetyl-piperazine amide group, respectively, showed comparable anti-migratory activity with 20% and 18%, respectively. The anti-proliferative effect of **16** on both cancer cell lines was moderate, compared to **15**, which lacks cytotoxic activity. On the other hand, the 3-aminoquinoline amide **24**, showed anti-migratory activity of 18% with GI_50_ above 50 µM on MDA-MB-231 cell line. When comparing Wiskostatin and carbazole derivatives from Table 1, compound **10** exhibited anti-proliferative activity with GI_50_ values comparable with Wiskostatin on both cancer cell lines. However, compound **10** showed higher anti-migratory potency with 13% at 2.1 µM, compared with 5% anti-migratory effect of Wiskostatin at 2 µM on MDA-MB-231 breast cancer cells. Therefore, compound **10** exhibits similar cytotoxicity with improved anti-migratory potential compared to Wiskostatin.

The Rho GTPases are believed to stimulate plasma membrane protrusion by inducing actin filament nucleation and polymerization on or close to membranes [43]. In particular, the Rho GTPase protein Cdc42, through activation of N-WASP/Arp2/3 pathway, is an important mediator of actin polymerization and filopodium extension [44,45]. Therefore, compounds that interfere with this process might be potentially useful molecular probes for the study of cell migration and invasion. To determine changes in actin cytoskeletal structures, we treated MDA-MB-231 breast cancer cells with vehicle or compounds **10**, **14**, **15**, and Wiskostatin at 10 µM for 24 h (Figure 4).

To identify F-actin based cell surface extensions, cells were stained with rhodamine phalloidin to localize F-actin. The results showed that untreated cells (control) demonstrated a strong formation of lamellipodia extensions, membrane ruffles, and stress fibers, with few filopodia. Wiskostatin and compounds **14** and **15** moderately reduced lamellipodia extensions when compared with control cells at concentrations that do not affect cell viability. In addition, compound **10**, at concentrations that inhibit 13% cell migration (2 µM), exhibited a marked reduction in lamellipodia formation when compared to vehicle, and in particular with Wiskostatin, which inhibits cell migration in 5% at 2 µM (Table 1). Compound **15** demonstrates a marked reduction in polymerized actin indicating inhibition of Arp2/3 mediated actin nucleation. Specific inhibition of filopodia, as would be predicted by WASP inhibition, could not be determined since the vehicle control cells exhibited more invadopodia and lamellipodia than filopodia. In general, these results suggest that new N-alkyl-3,6-dibromocarbazoles and compounds derivatives of Wiskostatin can be explored as new probes to study actin dynamics in cancer cells, or to further develop new anti-cancer and anti-metastatic drugs.

### 2.2. N-alkyl-5-Bromoindole Derivatives

**Scheme 2:**
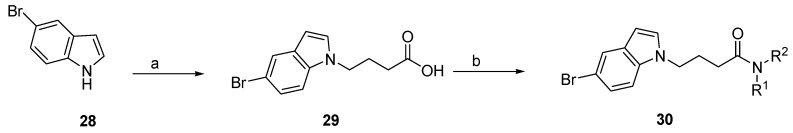
General synthetic procedure of 5-bromoindole-4-butyramide derivative **30**. Reagents and conditions: (**a**) (i) Ethyl 4-bromobutyrate, Cs_2_CO_3_, DMF, 100 °C, 16 h; (ii) KOH, DMF/water, 80 °C, 2-6 h, 80%; (**b**) HOBt, EDAC, CH_2_Cl_2_, Et_3_N, rt, amine: R-NH_2_ or HNR^1^R^2^, 2-8 h.

To further explore the N-alkyl-5-bromoindole butyramide derivative series, we synthesized several compounds in which the carbazole core was replaced by a 5-bromoindole ring (Scheme 2). The strategy is to analyze the effect of using a smaller ring system as a core connected to the C3 linker side chain and the amide group. The synthesis of N-alkyl-5-bromoindole derivatives is described in Scheme 2. The 5-bromoindole **28** was reacted with ethyl 4-bromobutyrate, followed by hydrolysis to yield the corresponding 5-bromoindole-4-butyric acid **29**. Since N-alkylation in indoles is more difficult than in carbazoles, the reaction rate to obtain **29** was improved by using Cs_2_CO_3_ as a base over K_2_CO_3_, where Cs_2_CO_3_ solubility is ten times higher in organic solvents than K_2_CO_3_. For the generation of 5-bromoindole-4-butyramide derivative **30**, carboxylic acid **29** was therefore used as starting material, which reacted with different amines via an amide coupling reaction using reaction conditions similar as described in Scheme 1 (see the Supplementary Materials for representative ^1^H and ^13^C NMR spectral data).

The in vitro anti-proliferative and anti-migratory activities of compounds **31–34** are represented in Table 2. In this assay, the replacement of the rigid carbazole ring core by an indole resulted in loss of cytotoxic activity on both MCF-7 and MDA-MB-231 cell lines. For example, from the four representative 5-bromoindole derivatives synthesized, it can be observed that only compound **34** showed moderate cytotoxic effect on MCF-7 cells with GI_50_ of 18.4 µM, while GI_50_ value on MDA-MB-231 breast cancer cells was above 50 µM. Furthermore, the remaining compounds in that series had GI_50_s above 50 µM in both breast cancer cell lines. Additionally, after 24 h treatment at 10 µM using the wound-healing assay in the MDA-MB-231 cell line, no migration inhibition could be observed. Hence, the absence of any activity of 5-bromoindole derivative series, together with the fact that several carbazole derivatives show promising activity, a structure-activity relationship (SAR) can be established. For example, it appears that the presence of a carbazole ring core, the C3 linker region, and amide group improve both anti-proliferative and anti-migratory activity.

## 3. Materials and Methods

### 3.1. General Methods

All experiments were carried out in pre-dried glassware (≥1 h, 80–90 °C) under a nitrogen atmosphere. Nuclear magnetic resonance (NMR) spectra were obtained using a 400 MHz Bruker Avance UltraShield™ spectrometer. ^1^H (400 MHz) and ^13^C (100 MHz) NMR were recorded in CDCl_3_ or DMSO-*d*_6_, unless otherwise used, and the chemical shift was expressed in parts per million (ppm) relative to CDCl3 (δ 7.26 for ^1^H and δ 77.0 for ^13^C) or DMSO-*d*_6_ (δ 2.50 for ^1^H and δ 39.5 for ^13^C) as the internal standard. ^1^H NMR data is reported as position (δ), relative integral, multiplicity (s, singlet; d, doublet; t, triplet; q, quartet; dt, doublet of triplets; dd, doublet of doublets; dq, doublet of quartets; m, multiplet; br, broad peak), coupling constant (*J*) in hertz (Hz), and the assignment of the atom. The shift in ppm for multiplets correspond to the centermost value of the entire splitting pattern. ^13^C NMR data are reported as position (δ) and assignment of the atom. Microwave reactions were conducted in a CEM Discovery Microwave for Drug Discovery, SP- 1445. High resolution electrospray ionization mass spectrometry (ESI-HRMS) data were obtained on a Thermo Scientific™ Q Exactive™ Hybrid Quadrupole-Orbitrap Mass Spectrometer with high performance liquid chromatography (HPLC) Agilent 1200 utilizing a Zorbax SB-C18 column (2.1 mm × 50 mm, 1.8µm) at 40 °C, and a mobile phase of acetonitrile containing 5% Milli Q water at a flow rate of 0.35 mL/min (run time: 6.5 min), and 1 µL injection volume.

### 3.2. Synthesis Methods

Progress of the reaction was monitored via TLC analysis using general purpose silica gel on glass 5 × 20 cm with UV indicator, 250 μm, 60 Å medium pore diameter, UV indicator, and visualized by UV fluorescent Spectroline E Series Ultraviolet lamps, in most cases followed by staining with I2. The compounds were purified via column chromatography over silica gel (70–230 mesh, 60 Å) with the appropriate size column (24/40, 12 in. × 0.5 in.) or (24/40, 12 in. × 0.72 in.). Wiskostatin compound was obtained from MilliporeSigma.

#### 3.2.1. General Procedure for the Synthesis of 4-(3,6-Dibromo-Carbazol-9-yl)-Butyric Acid (**2**)

A 50 mL three-neck round-bottom flask, equipped with a reflux condenser, was charged with 3,6-dibromocarbazole **1** (0.325 g, 1.0 mmol), K_2_CO_3_ (0.1382 g, 1 mmol), and ethyl 4-bromobutyrate 2 (0.4436 mL, 3.1 mmol), dissolved in DMF (5 mL). After 15 min of stirring at room temperature, the reaction mixture was refluxed at 80 °C for 2 h. After completion of the reaction (analyzed by TLC), water (1 mL) and KOH (1.0 mmol) was added and the reaction mixture, refluxed at 80 °C for 2 h. After the reaction was completed (analyzed by TLC), the mixture was allowed to reach room temperature. The mixture was washed with water (20 mL) and the product was extracted using dichloromethane (3 × 10 mL). The organic layer was washed with brine and dried with Na2SO4, and filtered and concentrated under reduced pressure. The crude oil was purified via column chromatography over silica gel and 50% ethyl acetate in hexane, and the product obtained as a white solid for the precursor 4-(3,6-Dibromo-carbazol-9-yl)-butyric acid **2** (0.3535 g, 0.86 mmol, 86%). TLC analysis in ethyl acetate-hexane (1:1), *R_f_* = 0.19. ^1^H NMR (400 MHz, CDCl_3_) δ 1.98 (2H, m), 2.27 (2H, t, *J* = 7.2 Hz), 4.41 (2H, t, *J* = 7.2 Hz), 7.30 (2H, d, *J* = 8.9 Hz), 7.56 (2H, dd, *J* = 2.0, 8.8 Hz), 8.14 (1H, d, *J* = 2.0 Hz); ^13^C NMR (100 MHz, CDCl_3_) 24.2, 31.1, 42.2, 111.8, 112.0, 123.4, 123.9, 139.5, 174.3. HR-FTMS (ESI) *m/z* calcd. for C_16_H_13_Br_2_NO_2_, [M + H]^+^ 411.9365, found 411.9365.

#### 3.2.2. Synthesis of 3,6-Dibromocarbazole-4-butyramide Derivatives (**4**–**27**)

#### 3.2.3. General Procedure for the Synthesis of 4-(3,6-Dibromocarbazol-9-yl)-N-(2-Morpholin-4-Ylethyl)Butyramide (**4**), and for Compounds **5**–**27**

A 50 mL three-neck round-bottom flask was charged with 4-(3,6-Dibromo-carbazol-9-yl)-butyric acid **2** (0.4111 g, 1.0 mmol), HOBT (0.2027 g, 1.5 mmol), and EDAC (0.2876 g, 1.5 mmol). The mixture was dissolved in CH_2_Cl_2_ (10 mL), stirred for 30 min, and 2-(4-morpholinyl)ethanamine **2** (0.133 g, 1.0 mmol) was added. After 15 min, Et_3_N (0.43 mL, 3.0 mmol) was added and the mixture was stirred at room temperature for 16 h. After completion of the reaction (analyzed by TLC), water was added (30 mL) and the product was extracted using dichloromethane (3 × 10 mL). The organic layer was washed with brine and dried with Na_2_SO_4_, and filtered and concentrated under reduced pressure. The crude oil was purified via column chromatography over silica gel and 10% methanol in dichloromethane, and the product obtained as a white solid (0.382 g, 0.73 mmol, 73%). TLC analysis in CH_2_Cl_2_-MeOH (9:1), *R_f_* = 0.26. ^1^H NMR (400 MHz, CDCl_3_) δ 2.0 (2H, m), 2.22 (2H, t, *J* = 6.8 Hz), 2.35 (2H, t, *J* = 4 Hz), 2.50 (4H, t, *J* = 1.6 Hz), 3.19 (2H, m), 3.56 (4H, t, *J* = 4.4 Hz), 4.39 (2H, t, *J* = 6.8 Hz), 7.60 (2H, d, *J* = 1.6 Hz), 8.0 (2H, bs), 8.46 (2H, bs); ^13^C NMR (100 MHz, CDCl_3_) 24.0, 31.2, 34.3, 41.8, 53.15, 53.2, 54.9, 57.2, 66.1, 111.3, 111.5, 122.9, 123.4, 128.8, 139.0, 161.0. HR-FTMS (ESI) *m/z* calcd. for C_22_H_25_Br_2_N_3_O_2_, [M + H]^+^ 524.0366, found 524.0367.

##### Synthesis of 4-(3,6-Dibromocarbazol-9-yl)-*N*-(2-methoxyethyl)butyramide (**5**)

The crude oil was purified via column chromatography over silica gel and 10% methanol in dichloromethane, and the product obtained as a white solid (0.096 g, 0.21 mmol, 63%). TLC analysis in CH_2_Cl_2_-MeOH (9:1), *R_f_* = 0.56. ^1^H NMR (400 MHz, CDCl_3_) δ 1.63 (2H, bs), 2.15 (2H, t, *J* = 6.0 Hz), 2.21 (2H, m), 3.63 (3H, s), 3.46 (2H, d, *J* = 2.0 Hz), 4.39 (2H, t, *J* = 6.4 Hz), 7.35 (2H, d, *J* = 8.8 Hz), 7.57 (2H, dd, *J* = 1.6, 8.8 Hz), 8.16 (2H, *d*, *J* = 1.6 Hz); ^13^C NMR (100 MHz, CDCl_3_) 24.1, 32.4, 39.2, 42.3, 58.7, 71.0, 110.5, 112.1, 123.2, 123.4, 129.1, 139.2, 171.6. HR-FTMS (ESI) *m*/*z* calcd. for C_19_H_20_Br_2_N2O_2_, [M + H]^+^ 468.9944, found 468.9947.

##### Synthesis of 4-(3,6-Dibromocarbazol-9-yl)-*N*-(3-imidazol-1-yl-propyl)butyramide (**6**)

The crude oil was purified via column chromatography over silica gel and 10% methanol in dichloromethane, and the product obtained as a white solid (0.078 g, 0.15 mmol, 49%). TLC analysis in CH_2_Cl_2_-MeOH (9:1), *R_f_* = 0.22. ^1^H NMR (400 MHz, CDCl_3_) δ 1.96 (2H, t, *J* = 6.8 Hz), 2.07 (2H, t, *J* = 6.4 Hz), 2.20 (2H, m), 3.23 (2H, q, *J* = 6.4 Hz), 3.98 (2H, t, *J* = 6.8 Hz), 4.38 (2H, t, *J* = 6.8 Hz), 6.93 (1H, bs), 7.05 (1H, Bs), 7.33 (2H, d, *J* = 8.4 Hz), 7.51 (1H, bs), 7.56 (2H, dd, *J* = 2.0, 8.8 Hz), 8.15 (2H, d, *J* = 1.6 Hz); ^13^C NMR (100 MHz, CDCl_3_) 24.0, 31.0, 32.3, 36.9, 42.2, 44.8, 110.5, 112.2, 112.3, 123.3, 123.4, 129.1, 129.3, 129.4, 139.3, 171.9. HR-FTMS (ESI) *m*/*z* calcd. for C_22_H_22_Br_2_N_4_O, [M + H]^+^ 519.0213, found 519.0211.

##### Synthesis of 4-(3,6-Dibromocarbazol-9-yl)-*N*-(2-piperidin-1-ylethyl)butyramide (**7**)

The crude oil was purified via column chromatography over silica gel and 10% methanol in dichloromethane, and the product obtained as a white solid (0.1033 g, 0.2 mmol, 59%). TLC analysis in CH_2_Cl_2_-MeOH (9:1), *R_f_* = 0.47. ^1^H NMR (400 MHz, CDCl_3_) δ 1.43 (2H, q, *J* = 4. 9 Hz), 1.52 (4H, m), 2.11 (4H, dt*, J* = 3.3, 11.7 Hz), 2.34 (3H, bs), 2.38 (3H, t, *J* = 6.0 Hz), 3.31 (2H, q, *J* = 5.5 Hz), 4.29 (2H, t, *J* = 6.4 Hz), 6.05 (1H, bs), 7.28 (2H, d, *J* = 8.7 Hz), 7.5 (2H, dd, *J* = 1.9, 8.7 Hz), 8.06 (2H, d, *J* = 1.9 Hz); ^13^C NMR (100 MHz, CDCl_3_) 24.0, 24.2, 32.2, 35.9, 42.2, 54.2, 57.0, 110.5, 112.0, 123.1, 123.2, 128.9, 139.2, 171.4. HR-FTMS (ESI) *m*/*z* calcd. for C_23_H_27_Br_2_N_3_O, [M + H]^+^ 522.0573, found 522.0572.

##### Synthesis of 4-(3,6-Dibromocarbazol-9-yl)-*N*-(2-piperazin-1-ylethyl)butyramide (**8**)

The crude oil was purified via column chromatography over silica gel and 10% methanol in dichloromethane, and the product obtained as a white solid (0.1425 g, 0.27 mmol, 71%). TLC analysis in CH_2_Cl_2_-MeOH (9:1), *R_f_* = 0.23. ^1^H NMR (400 MHz, CDCl_3_) δ 1.9 (1H, bs), 2.11 (2H, t, *J* = 6.4 Hz), 2.20 (2H, m), 2.38 (3H, bs), 2.42 (4H, t, *J* = 5.9 Hz), 2.84 (4H, t, *J* = 4.7 Hz), 3.32 (2H, q, *J* = 5.4 Hz), 4.38 (2H, t, *J* = 6.7 Hz), 7.33 (2H, d, *J* = 8.7 Hz), 7.54 (2H, dd, *J* = 1.9, 10.6 Hz), 8.14 (2H, s); ^13^C NMR (100 MHz, CDCl_3_) 24.0, 32.2, 35.6, 42.4, 45.9, 54.1, 57.0, 110.6, 112.1, 123.2, 123.5, 139.4, 171.4. HR-FTMS (ESI) *m*/*z* calcd. for C_23_H_27_Br_2_N_3_O, [M + H]^+^ 523.0526, found 523.0526.

##### Synthesis of 4-(3,6-Dibromocarbazol-9-yl)-*N*-(3-morpholin-4-ylpropyl)butyramide (**9**)

The crude oil was purified via column chromatography over silica gel and 10% methanol in dichloromethane, and the product obtained as a white solid (0.1496 g, 0.28 mmol, 69%). TLC analysis in CH_2_Cl_2_-MeOH (9:1), *R_f_* = 0.59. ^1^H NMR (400 MHz, CDCl_3_) δ 1.56 (2H, m, *J* = 6.0 Hz), 1.95 (2H, t, *J* = 6.8 Hz), 2.14 (4H, m), 2.23 (2H, bs), 2.36 (2H, t, *J* = 6.4 Hz), 3.25 (2H, q, *J* = 5.6 Hz), 3.42 (1H, bs), 4.31 (2H, t, *J* = 6.8 Hz), 7.31 (2H, d, *J* = 8.8 Hz), 7.54 (2H, dd, *J* = 2.0, 8.8 Hz), 8.12 (2H, d, *J* = 1.6 Hz); ^13^C NMR (100 MHz, CDCl_3_) 24.0, 24.4, 32.3, 39.6, 42.1, 53.5, 58.0, 66.8, 110.6, 112.1, 123.2, 123.4, 129.1, 139.3, 171.1. HR-FTMS (ESI) *m*/*z* calcd. for C_23_H_27_Br_2_N_3_O_2_, [M + H]^+^ 538.0522, found 538.0521.

##### Synthesis of 4-(3,6-Dibromocarbazol-9-yl)-*N*-(4-diethylamino-1-methylbutyl)butyramide (**10**)

The crude oil was purified via column chromatography over silica gel and 10% methanol in dichloromethane, and the product obtained as a white solid (0.0063 g, 0.040 mmol, 9.1%). TLC analysis in CH_2_Cl_2_-MeOH (9:1), *R_f_* = 0.21. ^1^H NMR (400 MHz, CDCl_3_) δ 0.89 (2H, m), 1.19 (6H, t, *J* = 7.6 Hz), 1.64 (2H, m), 2.20 (3H, bs), 2.25 (2H, t, *J* = 6.8 Hz), 2.62 (2H, t, *J* = 6.4 Hz), 3.29 (4H, m), 3.60 (2H, t, *J* = 7.20 Hz), 4.37 (2H, t, *J* = 7.20 Hz), 7.33 (2H, d, *J* = 8.8 Hz), 7.55 (2H, dd, *J* = 2.0, 8.8 Hz), 8.13 (2H, d, *J* = 2.0 Hz), 9.28 (1H, bs); ^13^C NMR (100 MHz, CDCl_3_) 8.5, 21.2, 21.3, 24.4, 29.7, 32.7, 33.6, 42.6, 44.6, 47.0, 52.3, 110.7, 112.0, 123.2, 123.5, 129.1, 139.4, 172.0. HR-FTMS (ESI) *m*/*z* calcd. for C_25_H_33_Br_2_N_3_O_2_, [M + H]^+^ 552.1043, found 552.1044.

##### Synthesis of 4-[4-(3,6-Dibromocarbazol-9-yl)-butyrylamino]piperidine-1-carboxylic acid ethyl ester (**11**)

The crude oil was purified via column chromatography over silica gel and 10% methanol in dichloromethane, and the product obtained as a white solid (0.1254 g, 0.22 mmol, 85.3%). TLC analysis in CH_2_Cl_2_-MeOH (9:1), *R_f_* = 0.63. ^1^H NMR (400 MHz, CDCl_3_) δ 1.25 (3H, t, *J* = 7.2 Hz), 1.88 (2H, dd, *J* = 2.8, 12.4 Hz), 2.08 (2H, t, *J* = 6.4 Hz), 2.19 (2H, m, *J* = 6.8 Hz), 2.88 (2H, t, *J* = 12.0 Hz), 3.92 (1H, m, *J* = 3.6 Hz), 4.12 (4H, q, *J* = 6.8 Hz), 4.37 (2H, t, *J* = 6.8 Hz), 5.16 (1H, d, *J* = 7.6 Hz), 7.32 (2H, d, *J* = 8.8 Hz), 7.55 (2H, dd, *J* = 2.0, 8.8 Hz), 8.14 (2H, d, *J* = 1.6 Hz); ^13^C NMR (100 MHz, CDCl_3_) 14.7, 24.0, 32.0, 32.4, 42.2, 42.7, 46.8, 61.4, 110.5, 112.2, 123.3, 123.5, 129.2, 139.3, 155.4, 170.1. HR-FTMS (ESI) *m*/*z* calcd. for C_24_H_27_Br_2_N_3_O_3_, [M + H]^+^ 566.0471, found 566.0470.

##### Synthesis of 4-(3,6-Dibromocarbazol-9-yl)-1-morpholin-4-ylbutan-1-one (**12**)

The crude oil was purified via column chromatography over silica gel and 10% methanol in dichloromethane, and the product obtained as a white solid (0.0768 g, 0.16 mmol, 52%). TLC analysis in CH_2_Cl_2_-MeOH (9:1), *R_f_* = 0.94. ^1^H NMR (400 MHz, CDCl_3_) δ 2.15 (4H, t, *J* = 6.0 Hz), 2.21 (2H, m, *J* = 6.8 Hz), 3.16 (2H, t, *J* = 4.8 Hz), 3.50 (2H, t, *J* = 4.4 Hz), 3.64 (4H, dt, *J* = 2.8, 6.4 Hz), 7.31 (2H, d, *J* = 8.8 Hz), 7.55 (2H, dd, *J* = 1.6, 8.4 Hz), 8.14 (2H, d, *J* = 1.6 Hz); ^13^C NMR (100 MHz, CDCl_3_) 23.6, 28.6, 41.5, 41.9, 45.1, 65.9, 66.0, 111.3, 111.5, 122.9, 123.41, 128.8, 139.0, 170.0. HR-FTMS (ESI) *m*/*z* calcd. for C_20_H_20_Br_2_N_2_O_2_, [M + H]^+^ 480.9944, found 480.9945.

##### Synthesis of 4-(3,6-Dibromocarbazol-9-yl)-1-thiomorpholin-4-ylbutan-1-one (**13**)

The crude oil was purified via column chromatography over silica gel and 10% methanol in dichloromethane, and the product obtained as a white solid (0.0712 g, 0.41 mmol, 35%). TLC analysis in CH_2_Cl_2_-MeOH (9:1), *R_f_* = 0.60. ^1^H NMR (400 MHz, CDCl_3_) δ 2.11 (2H, t, *J* = 6.3 Hz), 2.21 (2H, m), 2.34 (2H, t, *J* = 5.0 Hz), 2.62 (2H, t, *J* = 5.2 Hz), 3.46 (2H, t, *J* = 5.0 Hz), 3.89 (2H, t, *J* = 5.0 Hz), 4.41 (2H, t, *J* = 6.7 Hz), 7.32 (2H, d, *J* = 8.6 Hz), 7.56 (2H, dd, *J* = 1.9, 8.7 Hz), 8.15 (2H, d, *J* = 1.7 Hz); ^13^C NMR (100 MHz, CDCl_3_) 23.5, 27.3, 27.4, 29.0, 42.1, 44.3, 47.8, 110.6, 112.2, 123.3, 123.5, 129.1, 139.4, 169.9. HR-FTMS (ESI) *m*/*z* calcd. for C_20_H_20_Br_2_N_3_OS, [M + H]^+^ 496.9715, found 496.9713.

##### Synthesis of 1-[4-(3,6-Dibromocarbazol-9-yl)butyryl]piperidine-4-carbonitrile (**14**)

The crude oil was purified via column chromatography over silica gel and 10% methanol in dichloromethane, and the product obtained as a white solid (0.0775 g, 0.16 mmol, 44%). TLC analysis in CH_2_Cl_2_-MeOH (9:1), *R_f_* = 0.97. ^1^H NMR (400 MHz, CDCl_3_) δ 1.63 (2H, t, *J* = 5.6 Hz), 1.85 (1H, m), 2.15 (2H, m), 2.84 (2H, m), 3.14 (2H, dt, *J* = 5.2, 13.6 Hz), 3.35 (2H, dt, *J* = 6.0, 14.4 Hz), 3.63 (2H, dq, *J* = 4.4, 13.6 Hz), 3.76 (2H, dq, *J* = 3.2, 13.6 Hz), 4.41 (2H, m), 7.31 (2H, d, *J* = 8.4 Hz), 7.54 (2H, dd, *J* = 1.6, 8.4 Hz), 8.14 (2H, d, *J* = 1.6 Hz); ^13^C NMR (100 MHz, CDCl_3_) 23.4, 26.2, 28.1, 28.6, 28.7, 39.5, 42.1, 43.0, 110.6, 112.2, 120.5, 123.3, 123.5, 129.1, 139.4, 169.8. HR-FTMS (ESI) *m*/*z* calcd. for C_22_H_21_Br_2_N_3_O, [M + H]^+^ 504.0104, found 504.0104.

##### Synthesis of 4-(3,6-Dibromocarbazol-9-yl)-1-(4-phenylpiperazin-1-yl)butan-1-one (**15**)

The crude oil was purified via column chromatography over silica gel and 10% methanol in dichloromethane, and the product obtained as a white solid (0.2658 g, 0.27 mmol, 69%). TLC analysis in CH_2_Cl_2_-MeOH (9:1), *R_f_* = 0.53. ^1^H NMR (400 MHz, CDCl_3_) δ 1.55 (2H, bs), 2.22 (2H, m), 2.97 (2H, t, *J* = 5.3 Hz), 3.15 (2H, t, *J* = 4.9 Hz), 3.34 (2H, t, *J* = 4.5 Hz), 3.30 (2H, t, *J* = 4.7 Hz), 4.43 (2H, t, *J* = 3.4 Hz), 6.92 (2H, t, *J* = 7.9 Hz), 7.29 (3H, t, *J* = 7.9 Hz), 7.34 (2H, d, *J* = 8.7 Hz), 7.54 (2H, dd, *J* = 1.8, 8.6 Hz), 8.15 (2H, d, *J* = 1.7 Hz); ^13^C NMR (100 MHz, CDCl_3_) 23.6, 28.9, 41.6, 42.2, 45.1, 49.4, 49.5, 110.6, 112.1, 116.8, 120.7, 123.3, 123.5, 129.2, 129.3, 139.4, 150.9, 170.0. HR-FTMS (ESI) *m*/*z* calcd. for C_26_H_25_Br_2_N_3_O, [M + H]^+^ 556.0417, found 556.0417.

##### Synthesis of 1-(4-Acetylpiperazin-1-yl)-4-(3,6-dibromocarbazol-9-yl)butan-1-one (**16**)

The crude oil was purified via column chromatography over silica gel and 10% methanol in dichloromethane, and the product obtained as a white solid (0.0775 g, 0.15 mmol, 62%). TLC analysis in CH_2_Cl_2_-MeOH (9:1), *R_f_* = 0.67. ^1^H NMR (400 MHz, CDCl_3_) δ 1.57 (3H, s), 2.08 (2H, bs), 2.12 (2H, bs), 2.21 (2H, bs), 3.19 (2H, t, *J* = 5.3 Hz), 3.45 (2H, t, *J* = 6.2), 3.64 (2H, t, *J* = 5.0 Hz), 4.42 (2H, t, *J* = 6.6 Hz), 7.32 (2H, dd, *J* = 4.2, 8.7 Hz), 7.54 (2H, dd, *J* = 1.9, 8.7 Hz), 8.14 (2H, d, *J* = 1.8 Hz); ^13^C NMR (100 MHz, CDCl_3_) 21.4, 28.8, 31.0, 41.5, 42.0, 42.2, 44.8, 45.9, 110.5, 112.2, 123.4, 129.1, 139.3, 169.3, 170.4. HR-FTMS (ESI) *m*/*z* calcd. for C_22_H_23_Br_2_N_3_O_2_, [M + H]^+^ 522.0209, found 522.0209.

##### Synthesis of 4-[4-(3,6-Dibromocarbazol-9-yl)butyryl]piperazine-1-carboxylic acid tert-butyl ester (**17**)

The crude oil was purified via column chromatography over silica gel and hexane-ethyl acetate (1:1) as the mobile phase, and the product obtained as a white solid (0.1977 g, 0.4 mmol, 80%). TLC analysis in hexane-ethyl acetate (1:1), *R_f_* = 0.35. ^1^H NMR (400 MHz, CDCl_3_) δ 1.47 (9H, s), 2.20 (4H, m), 3.16 (2H, bs), 3.26 (2H, bs), 3.40 (2H, t, *J* = 5.2 Hz), 3.60 (2H, t, *J* = 4.4 Hz), 4.41 (2H, t, *J* = 6.4 Hz), 7.32 (2H, d, *J* = 8.4 Hz), 7.54 (2H, dd, *J* = 1.6, 8.4 Hz), 8.14 (2H, d, *J* = 2.0 Hz); ^13^C NMR (100 MHz, CDCl_3_) 23.5, 28.4, 29.0, 41.4, 42.2, 45.0, 80.4, 110.5, 112.2, 123.3, 123.5, 129.1, 139.4, 154.5, 170.2. HR-FTMS (ESI) *m*/*z* calcd. for C_25_H_29_Br_2_N_3_O_3_, [M + Na]^+^ 602.0447, found 602.0446.

##### Synthesis of 4-(3,6-Dibromocarbazol-9-yl)-1-piperazin-1-ylbutan-1-one (**18**)

The crude oil was purified via column chromatography over silica gel and 10% methanol in dichloromethane, and the product obtained as a white solid (0.0383 g, 0.080 mmol, 80%). TLC analysis in CH_2_Cl_2_-MeOH (9:1), *R_f_* = 0.38. ^1^H NMR (400 MHz, CDCl_3_) δ 1.25 (1H, s), 2.18 (4H, m), 2.68 (2H, t, *J* = 4.8 Hz), 2.84 (2H, t, *J* = 5.2 Hz), 3.16 (2H, t, *J* = 4.8 Hz), 3.61 (2H, t, *J* = 5.2 Hz), 4.41 (2H, t, *J* = 6.4 Hz), 7.32 (2H, d, *J* = 8.4 Hz), 7.54 (2H, dd*, J* = 2.0, 8.8 Hz), 8.14 (2H, d, *J* = 1.6 Hz); ^13^C NMR (100 MHz, CDCl_3_) 22.6, 23.6, 42.2, 42.7, 45.8, 46.0, 46.3, 110.6, 112.1, 123.3, 123.5, 129.1, 139.4, 170.0. HR-FTMS (ESI) *m*/*z* calcd. for C_20_H_21_Br_2_N_3_O, [M + H]^+^ 480.0104, found 480.0101.

##### Synthesis of 4-(3,6-Dibromocarbazol-9-yl)-1-[4-(2-morpholin-4-ylethyl)piperazin-1-yl]butan-1-one (**19**)

The crude oil was purified via column chromatography over silica gel and 10% methanol in dichloromethane, and the product obtained as a white solid (0.2036 g, 0.34 mmol, 76.4%). TLC analysis in CH_2_Cl_2_-MeOH (9:1), *R_f_* = 0.52. ^1^H NMR (400 MHz, CDCl_3_) δ 2.12 (2H, t, *J* = 6.0 Hz), 2.18 (2H, t, *J* = 7.2 Hz), 2.24 (2H, m), 2.44 (2H, t, *J* = 5.2 Hz), 2.48 (4H, t, *J* = 4.4 Hz), 3.17 (2H, t, *J* = 4.8 Hz), 3.63 (2H, t, *J* = 5.2 Hz), 3.70 (4H, t, *J* = 4.8 Hz), 4.41 (2H, t, *J* = 6.4 Hz), 7.28 (2H, d, *J* = 6.0 Hz), 7.37 (2H, dd, *J* = 6.4 Hz, 8.8 Hz), 8.15 (2H, d, *J* = 2.1 Hz); ^13^C NMR (100 MHz, CDCl_3_) 23.5, 28.7, 41.6, 42.1, 45.0, 53.2, 53.3, 54.1, 55.4, 56.3, 66.9, 110.7, 112.1, 123.3, 123.4, 129.2, 139.4, 169.8. HR-FTMS (ESI) *m*/*z* calcd. for C_26_H_32_Br_2_N_4_O_2_, [M + H]^+^ 593.0944, found 593.0945.

##### Synthesis of 2-{4-[4-(3,6-Dibromocarbazol-9-yl)-butyryl]piperazin-1-yl}-*N*-isopropylacetamide (**20**)

The crude oil was purified via column chromatography over silica gel and 10% methanol in dichloromethane, and the product obtained as a white solid (0.2273 g, 0.39 mmol, 87.3%). TLC analysis in CH_2_Cl_2_-MeOH (9:1), *R_f_* = 0.68. ^1^H NMR (400 MHz, CDCl_3_) δ 1.17 (6H, d, *J* = 6.6 Hz), 2.10 (2H, t, *J* = 6.3 Hz), 2.21 (2H, m), 2.26 (2H, t, *J* = 4.7 Hz), 2.47 (2H, t, *J* = 4.5 Hz), 3.15 (2H, t, *J* = 4.5 Hz), 3.64 (2H, t, *J* = 4.5 Hz), 4.10 (1H, m), 4.43 (2H, t, *J* = 6.6 Hz), 6.73 (1H, bs), 7.31 (2H, d, *J* = 8.7 Hz), 7.54 (2H, dd, *J* = 1.9, 8.7 Hz), 8.14 (2H, d, *J* = 1.9 Hz); ^13^C NMR (100 MHz, CDCl_3_) 22.8, 23.4, 28.7, 40.8, 41.6, 42.0, 45.0, 53.0, 53.1, 61.5, 110.64, 112.1, 123.3, 123.5, 129.2, 139.4, 168.4, 170.0. HR-FTMS (ESI) *m*/*z* calcd. for C_25_H_30_Br_2_N_4_O_2_, [M + H]^+^ 579.0788, found 579.0790.

##### Synthesis of 4-(3,6-Dibromocarbazol-9-yl)-1-[4-(2-dimethylaminoethyl)piperazin-1-yl]butan-1-one (**21**)

The crude oil was purified via column chromatography over silica gel and 10% methanol in dichloromethane, and the product obtained as a white solid (0.0228 g, 0.04 mmol, 7%). TLC analysis in CH_2_Cl_2_-MeOH (9:1), *R_f_* = 0.50. ^1^H NMR (400 MHz, CDCl_3_) δ 1.25 (6H, s), 2.12 (2H, t, *J* = 6.0 Hz), 2.19 (2H, m), 2.24 (4H, t, *J* = 5.6 Hz), 2.43 (4H, t, *J* = 4.8 Hz), 3.19 (2H, t, *J* = 4.4 Hz), 3.64 (2H, t, *J* = 4.4 Hz), 4.40 (2H, t, *J* = 6.4 Hz), 7.31 (2H, d, *J* = 8.8 Hz), 7.53 (2H, dd, *J* = 1.6, 8.4 Hz,), 8.21 (2H, d, *J* = 2.0 Hz); ^13^C NMR (100 MHz, CDCl_3_) 23.5, 28.7, 41.5, 42.1, 45.0, 45.6, 53.1, 53.3, 56.1, 56.5, 110.6, 112.1, 123.2, 123.4, 129.2, 139.4, 170.0. HR-FTMS (ESI) *m*/*z* calcd. for C_24_H_30_Br_2_N_4_O, [M + H]^+^ 551.0839, found 551.0842.

##### Synthesis of 4-(3,6-Dibromocarbazol-9-yl)-*N*-(4-morpholin-4-ylphenyl)butyramide (**22**)

The crude oil was purified via column chromatography over silica gel and 10% methanol in dichloromethane, and the product obtained as a white solid (0.0689 g, 0.12 mmol, 40%). TLC analysis in CH_2_Cl_2_-MeOH (9:1), *R_f_* = 0.80. ^1^H NMR (400 MHz, DMSO*-d_6_*) δ 2.05 (2H, t, *J* = 6.8 Hz), 2.30 (2H, m), 3.02 (4H, t, *J* = 4.8 Hz), 3.71 (4H, t, *J* = 4.4 Hz), 4.44 (2H, t, *J* = 6.8 Hz), 6.86 (2H, d, *J* = 8.8 Hz), 7.41 (2H, d, *J* = 8.8 Hz), 7.61 (2H, dd, *J* = 0.8, 8.8 Hz), 7.64 (2H, d, *J* = 8.4 Hz), 8.48 (2H, s), 9.62 (1H, s); ^13^C NMR (100 MHz, DMSO-*d_6_*) 24.1, 32.7, 42.0, 48.9, 66.1, 111.3, 111.6, 115.4, 120.3, 122.9, 123.4,128.8, 131.5, 139.0, 147.0, 169.7. HR-FTMS (ESI) *m/z* calcd. for C_26_H_25_Br_2_N_3_O_2_, [M + H]^+^ 572.0366, found 572.0365.

##### Synthesis of 4-(3,6-Dibromocarbazol-9-yl)-*N*-(3,4,5-trimethoxyphenyl)butyramide (**23**)

The crude oil was purified via column chromatography over silica gel and 10% methanol in dichloromethane, and the product obtained as a white solid (0.051 g, 0.10 mmol, 30%). TLC analysis in CH_2_Cl_2_-MeOH (9:1), *R_f_* = 0.17. ^1^H NMR (400 MHz, DMSO*-d_6_*) δ 2.08 (2H, m), 2.28 (2H, t, *J* = 7.2 Hz), 3.60 (3H, s), 3.71 (3H, s), 3.72 (3H, s), 4.46 (2H, t, *J* = 6.8 Hz), 6.90 (2H, s), 7.60 (2H, dd, *J* = 1.6, 8.4 Hz), 7.64 (2H, d, *J* = 8.8 Hz), 8.47 (2H, d, *J* = 1.6 Hz), 9.71 (1H, s); ^13^C NMR (100 MHz, DMSO-*d_6_*) 6.96, 24.0, 32.0, 42.0, 55.6, 60.0, 96.9, 111.3, 111.6, 122.9, 123.4, 128.8, 133.3, 135.2, 139.1, 152.6, 170.1. HR-FTMS (ESI) *m*/*z* calcd. for C_25_H_24_Br_2_N_2_O_4_, [M + H]^+^ 577.0155, found 577.0142.

##### Synthesis of 4-(3,6-Dibromocarbazol-9-yl)-*N*-quinolin-3-ylbutyramide (**24**)

The crude oil was purified via column chromatography over silica gel and 10% methanol in dichloromethane, and the product obtained as a white solid (0.0275 g, 0.052 mmol, 15%). TLC analysis in hexane-ethyl acetate (1:1), *R_f_* = 0.20. ^1^H NMR (400 MHz, DMSO*-d_6_*) δ 2.13 (2H, m), 2.44 (2H, t, *J* = 6.8 Hz), 4.50 (2H, t, *J* = 6.5 Hz), 7.41 (1H, t, *J* = 7.4 Hz), 7.54 (1H, t, *J* = 8.0 Hz), 7.62 (1H, t, *J* = 7.9 Hz), 7.67 (1H, d, *J* = 8.6 Hz), 7.71 (1H, d, *J* = 8.3 Hz), 7.92 (2H, t, *J* = 8.4 Hz), 7.98 (2H, d, *J* = 8.4 Hz), 8.47 (2H, s), 8.64 (1H, s), 8.82 (1H, s), 10.3 (1H, s); ^13^C NMR (100 MHz, DMSO-*d_6_*) 23.9. 32.9, 42.0, 109.6, 111.4, 111.7, 119.2, 122.0, 123.0, 123.5, 124.5, 127.0, 127.4, 127.6, 127.7, 127.8, 128.5, 128.9, 132.8, 139.1, 144.1, 144.5, 171.3. HR-FTMS (ESI) *m*/*z* calcd. for C_25_H_19_Br_2_N_3_O, [M + H]^+^ 537.9947, found 537.9946.

##### Synthesis of 4-(3,6-Dibromocarbazol-9-yl)-*N*-(1H-indol-6-yl)butyramide (**25**)

The crude oil was purified via column chromatography over silica gel and 10% methanol in dichloromethane, and the product obtained as a white solid (0.1152 g, 0.22 mmol, 38%). TLC analysis in hexane-ethyl acetate (1:1), *R_f_* = 0.50. ^1^H NMR (400 MHz, DMSO*-d_6_*) δ 2.08 (2H, m), 2.35 (2H, t, *J* = 7.1 Hz), 4.47 (2H, t, *J* = 7.0 Hz), 6.33 (1H, s), 6.97, (1H, d, *J* = 9.1 Hz), 7.24 (1H, s), 7.40 (1H, d, *J* = 8.4 Hz), 7.61 (2H, d, *J* = 7.2 Hz), 7.66 (2H, d, *J* = 8.7 Hz), 7.95 (1H, s), 8.49 (2H, s), 9.75 (1H, s); ^13^C NMR (100 MHz, DMSO-*d_6_*) 24.2, 32.9, 42.1, 100.9, 102.2, 111.3, 111.6, 112.3, 119.7, 123.0, 123.5, 123.7, 124.8, 128.9, 133.3, 135.9, 139.1. HR-FTMS (ESI) *m*/*z* calcd. for C_24_H_19_Br_2_N_3_O, [M + H]^+^ 525.9947, found 9946.

##### Synthesis of *N*-(3H-Benzoimidazol-5-yl)-4-(3,6-dibromocarbazol-9-yl)butyramide (**26**)

The crude oil was purified via column chromatography over silica gel and 10% methanol in dichloromethane, and the product obtained as a white solid (0.100 g, 0.20 mmol, 44%). TLC analysis in hexane-ethyl acetate (1:1), *R_f_* = 0.70. ^1^H NMR (400 MHz, DMSO*-d_6_*) δ 2.16 (2H, m, *J* = 7.2 Hz), 3.18 (2H, t, *J* = 7.6 Hz), 4.52 (2H, t, *J* = 7.2 Hz), 6.66 (1H, dd, *J* = 2.0, 8.8 Hz), 7.60 (1H, dd, *J* = 2.0, 8.8 Hz), 7.68 (1H, d, *J* = 8.8 Hz), 8.05 (1H, s), 8.47 (1H, dd, *J* = 1.6, 8.0 Hz); ^13^C NMR (100 MHz, DMSO-*d_6_*) 23.6, 32.0, 42.3, 97.2, 111.8, 112.0, 114.3, 117.2, 122.3, 123.5, 124.0, 129.4, 139.5, 141.0, 141.2, 151.7, 173.0. HR-FTMS (ESI) *m*/*z* calcd. for C_23_H_18_Br_2_N_4_O, [M + H]^+^ 526.9900, found 526.9904.

##### Synthesis of 4-(3,6-Dibromocarbazol-9-yl)-*N*-(4-methoxyphenyl)butyramide (**27**)

The crude oil was purified via column chromatography over silica gel and 10% methanol in dichloromethane, and the product obtained as a white solid (0.1830 g, 0.35 mmol, 94%). TLC analysis in hexane-ethyl acetate (1:1), *R_f_* = 0.64. ^1^H NMR (400 MHz, DMSO*-d_6_*) δ 2.05 (2H, m), 2.30 (2H, t, *J* = 7.2 Hz), 3.70 (3H, s), 4.45 (2H, t, *J* = 7.2 Hz), 6.84 (2H, d, *J* = 9.2 Hz), 7.44 (2H, d, *J* = 9.2 Hz), 7.60 (2H, dd, *J* = 2.0, 8.8 Hz), 7.65 (2H, d, *J* = 8.8 Hz), 8.48 (2H, d, *J* = 2.0 Hz), 9.68 (1H, s); ^13^C NMR (100 MHz, DMSO-*d_6_*) 24.1, 32.7, 42.0, 55.1, 111.3, 111.6, 113.8, 120.7, 123.0, 123.5, 128.7, 132.3, 139.1, 155.1, 169.9. HR-FTMS (ESI) *m*/*z* calcd. for C_23_H_20_Br_2_N_2_O_2_, [M + H]^+^ 516.9944, found 516.9941.

#### 3.2.4. Synthesis of N-Alkyl-5-Bromoindole Derivatives (**31**–**34**)

#### 3.2.5. General Procedure for the Synthesis of 4-(5-Bromoindol-1-yl)Butyric Acid (**29**)

A 50 mL three-neck round-bottom flask, equipped with a reflux condenser, was charged with 5-bromoindole **28** (0.1960 g, 1.0 mmol), Cs_2_CO_3_ (0.9770 g, 3 mmol) and ethyl 4-bromobutyrate (0.43 mL, 3.0 mmol), dissolved in DMF (3 mL). After 15 min of stirring at room temperature, the reaction mixture was refluxed at 100 °C for 16 h. After completion of the reaction (analyzed by TLC), water (1 mL) and KOH (1.0 mmol) were added and the reaction mixture refluxed at 80 °C for 2 h. After the reaction was completed (analyzed by TLC), the mixture was allowed to reach room temperature and 1N HCl solution was added until reach neutral pH. The mixture was washed with water (20 mL) and the product was extracted using dichloromethane (3 × 10 mL). The organic layer was washed with brine and dried with Na_2_SO_4_, and filtered and concentrated under reduced pressure. The crude oil was purified via column chromatography over silica gel and 10% CH_2_Cl_2_ in MeOH, and the product obtained as a white solid for the precursor 4-(5-Bromoindol-1-yl)Butyric acid **29** (0.2260 g, 0.801 mmol, 80%). TLC analysis in CH_2_Cl_2_-MeOH (9:1), *R_f_* = 0.25. ^1^H NMR (400 MHz, CDCl_3_) δ 2.16 (2H, m), 2.34 (2H, t, *J* = 7.2 Hz), 4.20 (2H, t, *J* = 6.80 Hz), 6.44 (1H, d, *J* = 2.4 Hz), 7.08 (1H, 2, *J* = 3.2 Hz), 7.22 (1H, d, *J* = 6.4 Hz), 7.29 (1H, dd, *J* = 2.0, 8.8 Hz), 7.74 (1H, d, *J* = 1.6 Hz); ^13^C NMR (100 MHz, CDCl_3_) 25.1, 30.5, 45.3, 101.1, 110.7, 112.8, 123.5, 124.5, 128.9, 130.3, 134.6, 177.4. HR-FTMS (ESI) *m/z* calcd. for C_12_H_12_BrNO_2_, [M + H]^+^ 284.0104, found 284.0102.

#### 3.2.6. General Procedure for the Synthesis of 4-(5-Bromoindol-1-yl)-N-(3-Morpholin-4-ylpropyl)Butyramide (**31**), and for Compounds **32**–**34**

A 50 mL three-neck round-bottom flask was charged with 4-(5-Bromoindol-1-yl)Butyric acid **29** (0.098 g, 0.35 mmol), HOBT (0.2027 g, 1.5 mmol), and EDAC (0.2876 g, 1.5 mmol). The mixture was dissolved in DMF (5 mL), stirred for 30 min, and 3-morpholinopropylamine (0.0512 mL, 0.35 mmol) was added. After 15 min, Et_3_N (0.43 mL, 3.0 mmol) was added and the mixture stirred at room temperature for 16 h. After the reaction was completed (analyzed by TLC), water was added (30 mL) and the product was extracted using dichloromethane (3 × 10 mL). The organic layer was washed with brine and dried with Na_2_SO_4_, and filtered and concentrated under reduced pressure. The crude oil was purified via column chromatography over silica gel and 10% methanol in dichloromethane, and the product obtained as a white solid (0.0748 g, 0.18 mmol, 53%). TLC analysis in hexane-ethyl acetate (1:1), *R_f_* = 0.36. ^1^H NMR (400 MHz, CDCl_3_) δ 1.62 (2H, m), 2.01 (2H, t, *J* = 6.8 Hz), 2.21 (2H, m), 2.35 (4H, bs), 2.42 (2H, t, *J* = 6.4 Hz), 3.32 (2H, q, *J* = 5.6 Hz), 3.47 (4H, bs), 4.39 (2H, t, *J* = 6.8 Hz), 7.0 (1H, bs), 7.26 (1H, d, *J* = 1.2 Hz), 7.32 (1H, d, *J* = 8.8 Hz), 7.55 (1H, dd, *J* = 2.0, 8.8 Hz), 8.14 (1H, d, *J* = 1.6 Hz); ^13^C NMR (100 MHz, CDCl_3_) 24.5, 25.8, 32.6, 39.6, 45.2, 53.6, 58.0, 67.0, 101.1, 110.9, 112.6, 123.4, 124.3, 128.8, 130.2, 134.9, 171.2. HR-FTMS (ESI) *m/z* calcd. for C_19_H_26_BrN_3_O_2_, [M + H]^+^ 410.1261, found 410.1257.

##### Synthesis of 1-[4-(5-Bromoindol-1-yl)butyryl]piperidine-4-carbonitrile (**32**)

The crude oil was purified via column chromatography over silica gel and 10% methanol in dichloromethane, and the product obtained as a white solid (0.0356 g, 0.10 mmol, 24.4%). TLC analysis in CH_2_Cl_2_-MeOH (9:1), *R_f_* = 0.76. ^1^H NMR (400 MHz, DMSO-*d_6_*) δ 1.58 (4H, m), 1.81 (2H, bs), 1.94 (2H, t, *J* = 7.2 Hz), 2.26 (2H, t, *J* = 7.2 Hz), 3.07 (1H, m), 3.50 (2H, dt, *J* = 4.8, 15.2 Hz), 3.78 (2H, dt, *J* = 4.8, 13.6Hz), 4.18 (2H, t, *J* = 6.8Hz), 6.42 (1H, d, *J* = 3.2 Hz), 7.24 (1H, dd, *J* = 2.0, 8.4 Hz), 7.41 (1H, d, *J* = 3.2 Hz), 7.47 (1H, d, *J* = 8.8 Hz), 7.73 (1H, d, *J* = 2.0 Hz); ^13^C NMR (100 MHz, DMSO-*d_6_*) 22.7, 25.3, 28.2, 29.4, 29.7, 39.5, 43.1, 45.3, 101.0, 111.0, 112.7, 120.6, 123.5, 124.4, 128.9, 130.2, 134.9, 169.9. HR-FTMS (ESI) *m/z* calcd. for C_18_H_20_BrN_3_O, [M + H]^+^ 376.0842, found 376.0837.

##### Synthesis of 4-(5-Bromoindol-1-yl)-N-(3-imidazol-1-yl-propyl)butyramide (**33**)

The crude oil was purified via column chromatography over silica gel and 10% methanol in dichloromethane, and the product obtained as a white solid (0.1255 g, 0.32 mmol, 57%). TLC analysis in CH_2_Cl_2_-MeOH (9:1), *R_f_* = 0.44. ^1^H NMR (400 MHz, CDCl_3_) δ 1.81 (2H, m, *J* = 6.8 Hz), 1.98 (2H, m, *J* = 6.8 Hz), 2.06 (2H, t, *J* = 6.4 Hz), 3.01 (2H, q, *J* = 5.6 Hz), 3.95 (2H, t, *J* = 6.8 Hz), 4.17 (2H, t, *J* = 7.2 Hz), 6.42 (1H, d, *J* = 2.8 Hz), 6.95 (1H, bs), 7.23 (2H, dd, *J* = 1.6, 8.4 Hz), 7.40 (1H, d, *J* = 2.8 Hz), 7.45 (1H, d, *J* = 8.8 Hz), 7.72 (1H, d, *J* = 2.0 Hz), 7.90 (1H, t, *J* = 5.2 Hz); ^13^C NMR (100 MHz, CDCl_3_) 25.7, 31.0, 32.2, 36.5, 44.2, 45.6, 101.0, 111.0, 112.5, 119.1, 123.3, 124.4, 128.7, 128.8, 130.2, 134.7, 136.9, 172.4. HR-FTMS (ESI) *m/z* calcd. for C_18_H_21_BrN_4_O, [M + H]^+^ 391.0951, found 391.0956.

##### Synthesis of 4-(5-Bromoindol-1-yl)-N-(2-piperidin-1-ylethyl)butyramide (**34**)

The crude oil was purified via column chromatography over silica gel and 10% methanol in dichloromethane, and the product obtained as a white solid (0.1967 g, 0.50 mmol, 50%). TLC analysis in CH_2_Cl_2_-MeOH (9:1), *R_f_* = 0.47. ^1^H NMR (400 MHz, DMSO-*d_6_*) δ 1.35 (2H, m, *J* = 5.2 Hz), 1.47 (4H, m, *J* = 5.6 Hz), 1.96 (2H, m, *J* = 6.4 Hz), 2.01 (2H, t, *J* = 6.0 Hz), 2.30 (2H, t, *J* = 6.8 Hz), 2.35 (2H, bs), 3.13 (2H, q, *J* = 6.4 Hz), 4.18 (2H, t, *J* = 6.4 Hz) 7.35 (1H, dd, *J* = 2.0, 8.8 Hz), 7.54 (2H, d, *J* = 2.4 Hz), 7.56 (1H, s), 7.68 (1H, s), 7.71 (1H, t, *J* = 5.6 Hz); ^13^C NMR (100 MHz, DMSO-*d_6_*) 24.1, 25.5, 25.7, 32.4, 35.7, 45.8, 54.3, 57.2, 100.8, 109.4, 111.0, 118.5, 120.3, 128.9, 129.0, 134.7, 171.7. HR-FTMS (ESI) *m/z* calcd. for C_25_H_30_Br_2_N_4_O_2_, [M + H]^+^ 394.1312, found 394.1313.

### 3.3. Biological Evaluation

#### 3.3.1. Cell Culture

MCF-7 and MDA-MB-231 cells were cultured in Minimum Essential Medium Eagle (MEME) 10% FBS supplemented with Earle’s Balanced Salt Solution (EBSS), Non-essential Amino Acids (NEAA), Sodium Pyruvate, Pen/Strep, and L-glutamine 37 °C in 5% CO_2_.

#### 3.3.2. Sulforhodamine B (SRB) Assay

A stock solution of compounds was prepared at 50 mM in 100% DMSO. For cell preparation, a flask of 75 cm^2^ or 25 cm^2^ was used for 2.6 × 10^5^ cells/mL or 1.44 × 10^5^ cells/mL, respectively, with an 80–90% confluence. Cells were washed with PBS and trypsinized. The concentration of cells was determined using a 1:2 dilution with Trypan Blue and a hemocytometer. After cell count, the concentration was adjusted to have a 7.0–10.0 × 10^4^ cells/mL. Approximately 100 μL of cells suspension, compounds, control positive and control negative were added in triplicates to a 96 well plate. The positive control used was doxorubicin, and the negative control was DMSO 0.1%. All compounds at 50, 25, 12.5, 6.3, and 1.6 μM were incubated with cells at 37 °C for 48 hrs. For fixation, cold TCA 50% was used and incubated at 4 °C for 1 hr. Wells were washed and dried prior to tincture with 100 μL of SRB 0.4%. To remove excess SRB, acetic acid was used. For analysis, TRIS-Base Solution (pH = 10.5) was used and shaken prior to reading using an ELISA reader at 540 nm and the software SoftMax Pro 4.8. For each compound, 50% growth inhibition (GI_50_) was calculated from sigmoidal dose-response curves (variable-slope) that were generated with data obtained from experiments carried out in triplicates (GI_50_ values were generated with GraphPad Prism V. 6.02, GraphPad Software, Inc.).

#### 3.3.3. Wound Healing Assay (Scratch Method) Using MDA-MB-231 Cancer Cells

Prior to assays, cells were grown until 80–90% confluence was observed. We used a 75 cm^2^ flask for 2.6 × 10^5^ cells/mL in 10 mL, and for a 25 cm^2^ flask 1.44 × 10^5^ cells/mL in 5 mL. The cells were washed with PBS to remove all traces of FBS. We added trypsin at 2 mL for a 25 cm^2^ flask or 4 mL for a 75 cm^2^ flask, and incubated 5–10 min at 37 °C. At the end of the incubation time, cells were re-suspended and counted with hemocytometer using 1:2 dilutions with Trypan Blue. Subsequently, cell viability was calculated. In a 12 multiwell plate. Cells were seeded at 1.5–2.2 × 10^5^ cells/mL in 1 mL and incubated for 24 h. Cells were then rinsed with PBS and incubated in starving media (0.5% FBS) overnight. All controls and drugs were tested in triplicate. The vehicle control for each drug was prepared according to the drug’s DMSO concentration. Drugs were diluted and the final concentration at each well was 10 µM (or GI_50_/5 on MDA-MB-231 cells). The wound was made using a sterile pipette tip of 200 μL. Cells were then rinsed very gently with media without FBS and media with vehicle controls or drugs were added. After a 24 h incubation, the gap distance was evaluated using the software Lumera Infinity Analyze 6.4.0. Pictures were taken at 0, 8, 12, and 24 h using a 10X objective in an Inverted Laboratory Microscope Leica DM IL LED, and an Infinity1-3 3.1 Megapixel USB 2.0 camera CMOS. The percentage of migration was calculated using the following formula:

100 − [ (X₀⁄Ẍ₀)]*100 for time 0 h measurements

100 − [ (X₂₄⁄Ẍ₀)]*100 for time 24 h measurements

#### 3.3.4. Actin Polymerization Assay

MDA-MB-231 cells were treated with vehicle or compound derivatives at 10 μM (or at concentrations that do not affect cell viability) for 24 h at 37 °C in 5% CO_2_. Cells were fixed in 3.7% of formaldehyde, permeabilized using 0.2% Triton, and stained with Rhodamine Phalloidin to visualize F-actin. Fluorescence micrographs were acquired at 60x in an Eclipse E400 fluorescence microscope using a DS-Qi2 monochrome digital camera from Nikon for fluorescence imaging.

## 4. Conclusions

In this study, a new series of *N*-alkyl-3,6-dibromocarbazole and *N*-alkyl-5-bromoindole derivatives were designed, synthesized, and biologically evaluated for their anti-cancer and anti-migration effect on MCF-7 and MDA-MB-231 cancer cells. In addition, the effect of compounds on the actin cytoskeleton of MDA-MB-231 cancer cells was explored. From the results, we have established the Structure-Activity Relationships of novel derivatives of the lead compound Wiskostatin. We found that the carbazole moiety is needed for in vitro anti-cancer and anti-migratory potency. The replacement of the carbazole by an indole ring resulted in loss of activity. Moreover, the aliphatic or aromatic amide group was extended 3-carbon atoms away from the carbazole moiety. The most potent anti-proliferative compounds were those bearing a 3,6-dibromocarbazole group with GI_50_ values between 7–32 µM and 4.7–23 µM on MCF-7 and MDA-MB-231 cancer cells, respectively. The most promising results in terms of anti-migratory effect was exhibited by carbazole derivatives **10**, **23**, **14–16**, and **24** with migration inhibition in the range of 10–20% on the highly metastatic breast cancer cells MDA-MB-231. Future studies will improve these derivatives and also test their effects at higher concentrations and shorter times of migration to reduce potential cytotoxic effects, which may confound inhibition of cell migration. The carbazole derivatives **10**, **14**, and **15** were further examined as actin polymerization inhibitors and results demonstrated reduction in actin polymerization and extension of F-actin based structures. Further studies of mechanism of actions focused on the effect of these novel compounds in the Cdc42/N-WASP/Arp2/3 pathway is needed to fully characterize and analyze the potential of this new series of compounds as in vitro and in vivo anti-metastatic drugs.

## Figures and Tables

**Table 1:** Cell growth inhibition and anti-migration activity of 3,6-dibromocarbazole derivatives.

Comp.	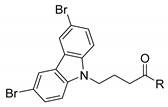 R =	GI_50_ (µM)^a^		Migration (%)^b,c^
		MCF-7	MDA-MB-231	
**4**	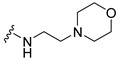	>50	>50	99 ± 6.03
**5**	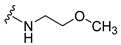	>50	>50	99 ± 5.98
**6**	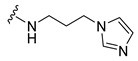	16.8	16	94 ± 3.93
**7**	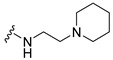	6.8	10	99 ± 0.03 (at 2 μM)
**8**	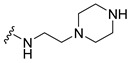	8	4.7	97 ± 4.90 (at 1 μM)
**9**	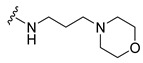	13.4	15.4	99 ± 1.58 (at 3.1 μM)
**10**	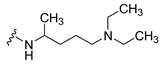	8.1	10.5	87 ± 4.65 (at 2.1 μM)
**11**	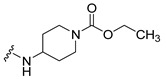	>50	>50	99 ± 0.02
**12**	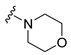	>50	>50	97 ± 6.41
**13**	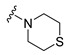	>50	25	99 ± 0.03
**14**	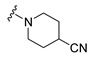	11.8	16.7	81 ± 8.96
**15**	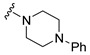	>50	>50	80 ± 5.87
**16**	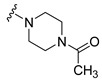	18.2	23	82 ± 7.52
**17**	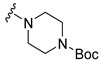	17.5	>50	99 ± 2.09
**18**	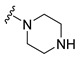	7.5	6.7	99 ± 0.02
**19**	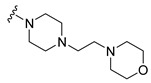	12.4	>50	99 ± 0.97
**20**	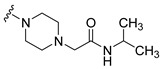	9.1	13.4	99 ± 0.05 (at 2.7 μM)
**21**	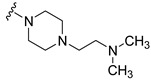	6.5	8	99 ± 0.48 (at 1.6 μM)
**22**	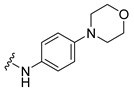	>50	>50	97 ± 4.19
**23**	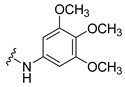	>50	19	90 ± 6.60
**24**	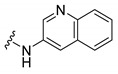	32.2	>50	82 ± 5.19
**25**	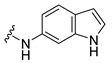	>50	>50	96 ± 4.69
**26**	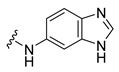	>50	>50	99 ± 6.52
**27**	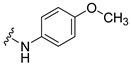	>50	>50	99 ± 0.05
**Wiskostatin**		9.7	8.3	95 ± 6.79 (at 2 μM)

^a^ GI_50_ = compound concentration required to inhibit MDA-MB-231 proliferation by 50% after 48 h treatment. Values are expressed as the mean of triplicate experiments, and standard deviation (SD) is <10%. ^b^ After 24 h, MDA-MB-231 cellular migration was determined by measuring the distance traveled from the edge of the scratch toward the center of the scratch, relative to control. ^c^ Percent relative migration values at 10 µM (or at concentrations that do not affect cell viability). Results are presented as means ± SD of three independent experiments.

**Table 2:** Cell growth inhibition and anti-migration activity of 5-bromoindole derivatives.

Comp.	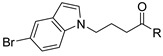 R =	GI_50_ (µM)^a^		Migration (%)^b,c^
		MCF-7	MDA-MB-231	
**31**	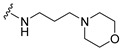	>50	>50	99 ± 0.05
**32**	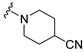	>50	>50	99 ± 7.78
**33**	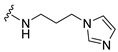	>50	>50	99 ± 0.02
**34**	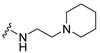	18.4	>50	99 ± 6.60

^a^ GI_50_ = compound concentration required to inhibit MDA-MB-231 proliferation by 50% after 48 h treatment. Values are expressed as the mean of triplicate experiments, and standard deviation (SD) is <10%. ^b^ After 24 h, MDA-MB-231 cellular migration was determined by measuring the distance traveled from the edge of the scratch toward the center of the scratch, relative to control. ^c^ Percent relative migration values at 10 µM. Results are presented as means ± SD of three independent experiments.

## Supplementary Materials

The following are available online. ^1^H and ^13^C NMR spectral data for representative compounds. Figure S1: ^1^H NMR Spectral Data of 2; Figure S2: ^13^C NMR Spectral Data of 2; Figure S3: ^1^H NMR Spectral Data of 7; Figure S4: ^13^C NMR Spectral Data of 7; Figure S5: ^1^H NMR Spectral Data of 12; Figure S6: ^13^C NMR Spectral Data of 12; Figure S7: ^1^H NMR Spectral Data of 14; Figure S8: ^13^C NMR Spectral Data of 14; Figure S9: ^1^H NMR Spectral Data of 16; Figure S10: ^1^H NMR Spectral Data of 22; Figure S11: ^13^C NMR Spectral Data of 22; Figure S12: ^1^H NMR Spectral Data of 24; Figure S13: ^13^C NMR Spectral Data of 24; Figure S14: ^1^H NMR Spectral Data of 29; Figure S15: ^13^C NMR Spectral Data of 29; Figure S16: ^1^H NMR Spectral Data of 33; Figure S17: ^13^C NMR Spectral Data of 33.
