# Supplementary material for: Synthesis, Anti-Cancer and Anti-Migratory Evaluation of 3,6-Dibromocarbazole and 5-Bromoindole Derivatives
Source: Molecules. 2019 Jul 24;24(15):2686. doi: 10.3390/molecules24152686 (PMC6696445; doi:10.3390/molecules24152686)
Supplement: Supplementary file 1 [file molecules-24-02686-s001.pdf]

-Supplementary Material-

Synthesis, anti-cancer and anti-migratory evaluation of 3,6-dibromocarbazole and 5-bromoindole derivatives

Krystal M. Butler-Fernández[1], Zulma Ramos[1], Adela M. Francis-Malavé[2], Joseph Bloom[1], Suranganie Dharmawardhane[3] and Eliud Hernández[1,*]

[a]*Department of Pharmaceutical Sciences, University of Puerto Rico, School of Pharmacy, San Juan, PR 00936*

[c]*Department of Biology, College of Natural Sciences, University of Puerto Rico, San Juan, PR 00931*

[b]*Department of Biochemistry, University of Puerto Rico, School of Medicine, San Juan, PR 00936*

* Correspondence: eliud.hernandez@upr.edu; Tel.: +1 (787) 758-2525, Ext. 5436 (P.R.)

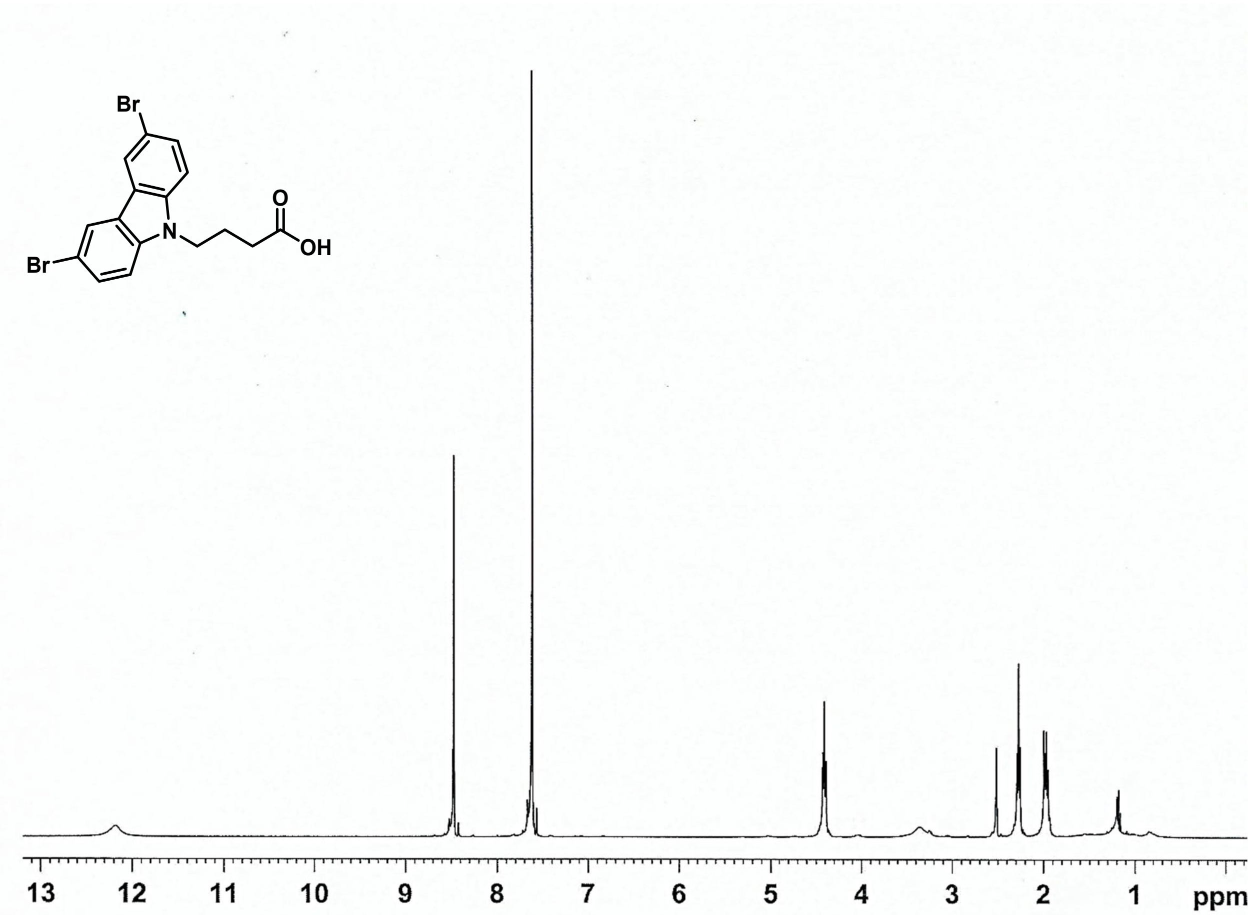

Figure S1: ^{1}H NMR Spectral Data of **2**

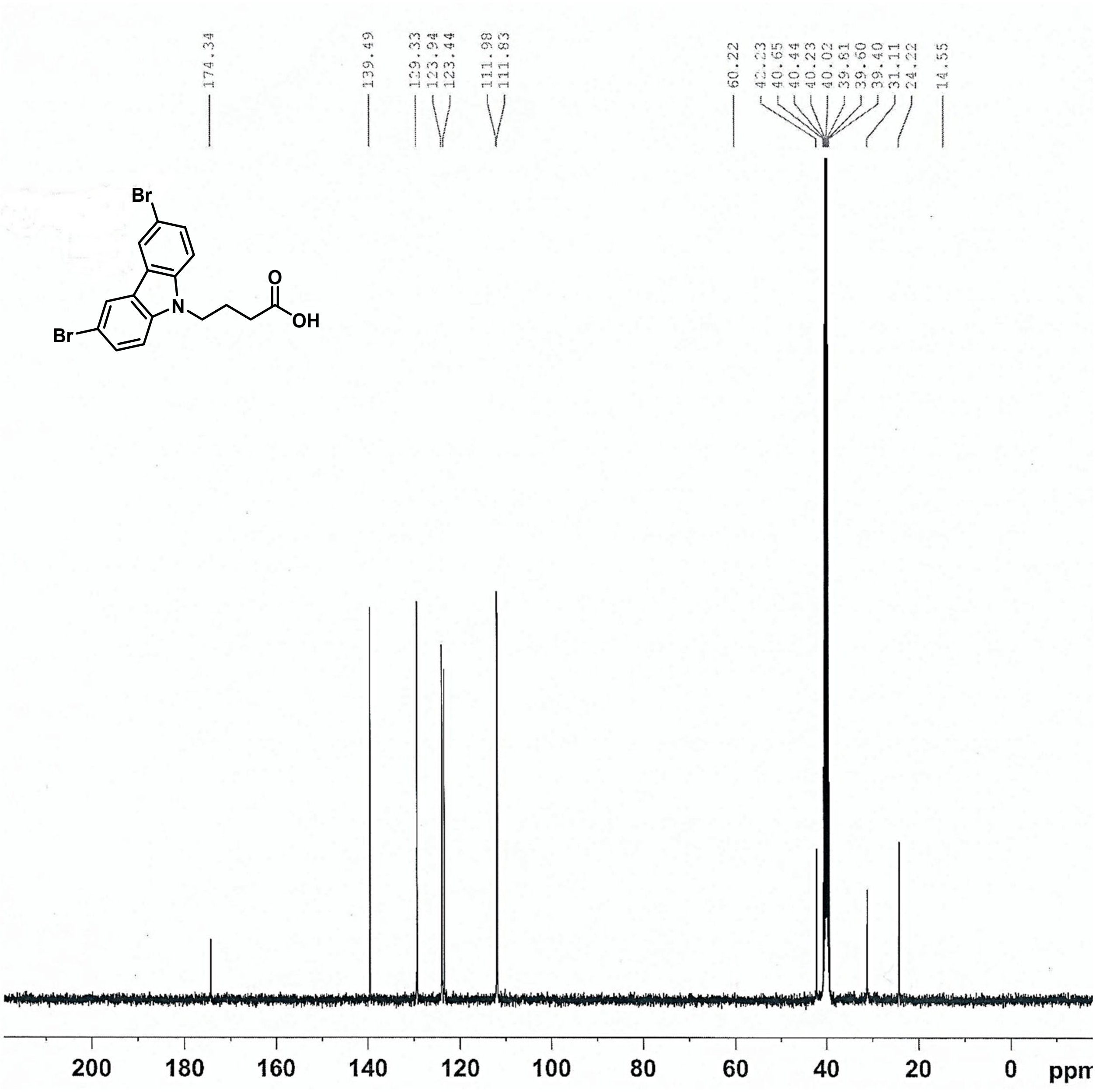

Figure S2: ^{13}C NMR Spectral Data of **2**

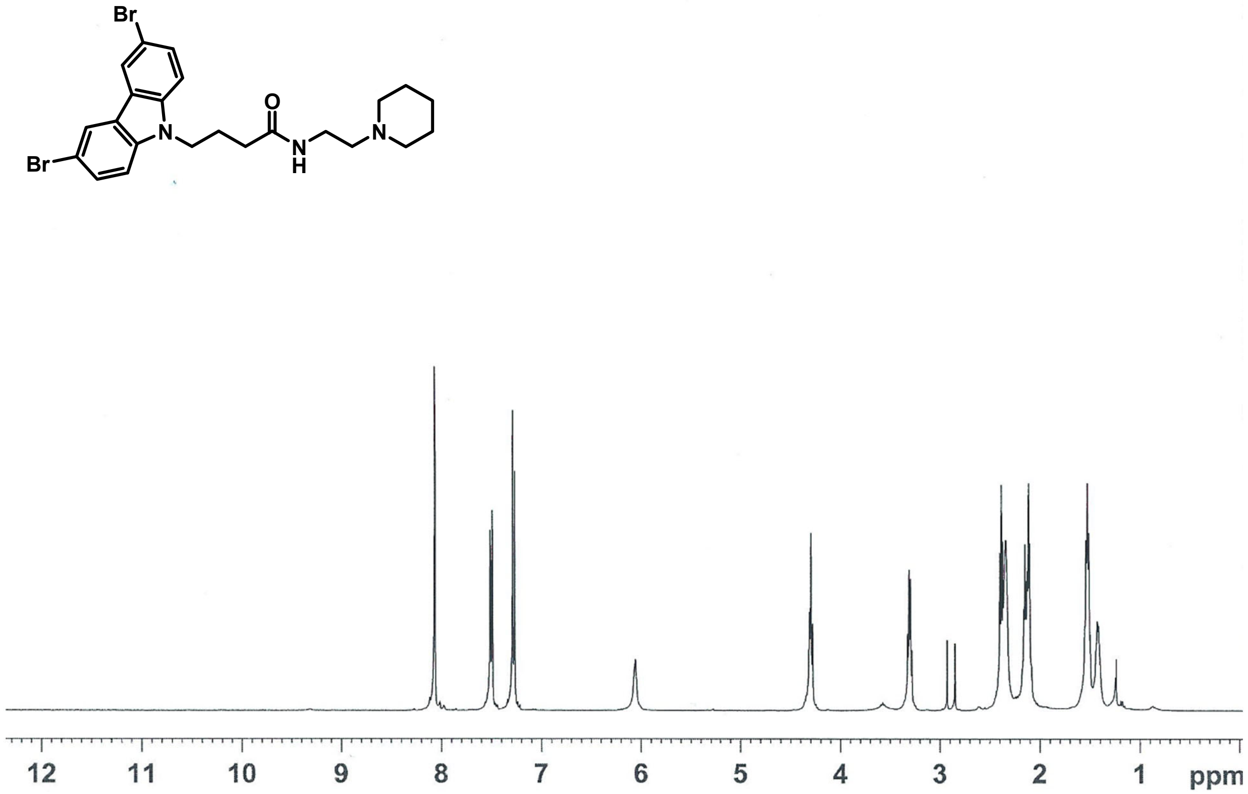

Figure S3: ^{1}H NMR Spectral Data of **7**

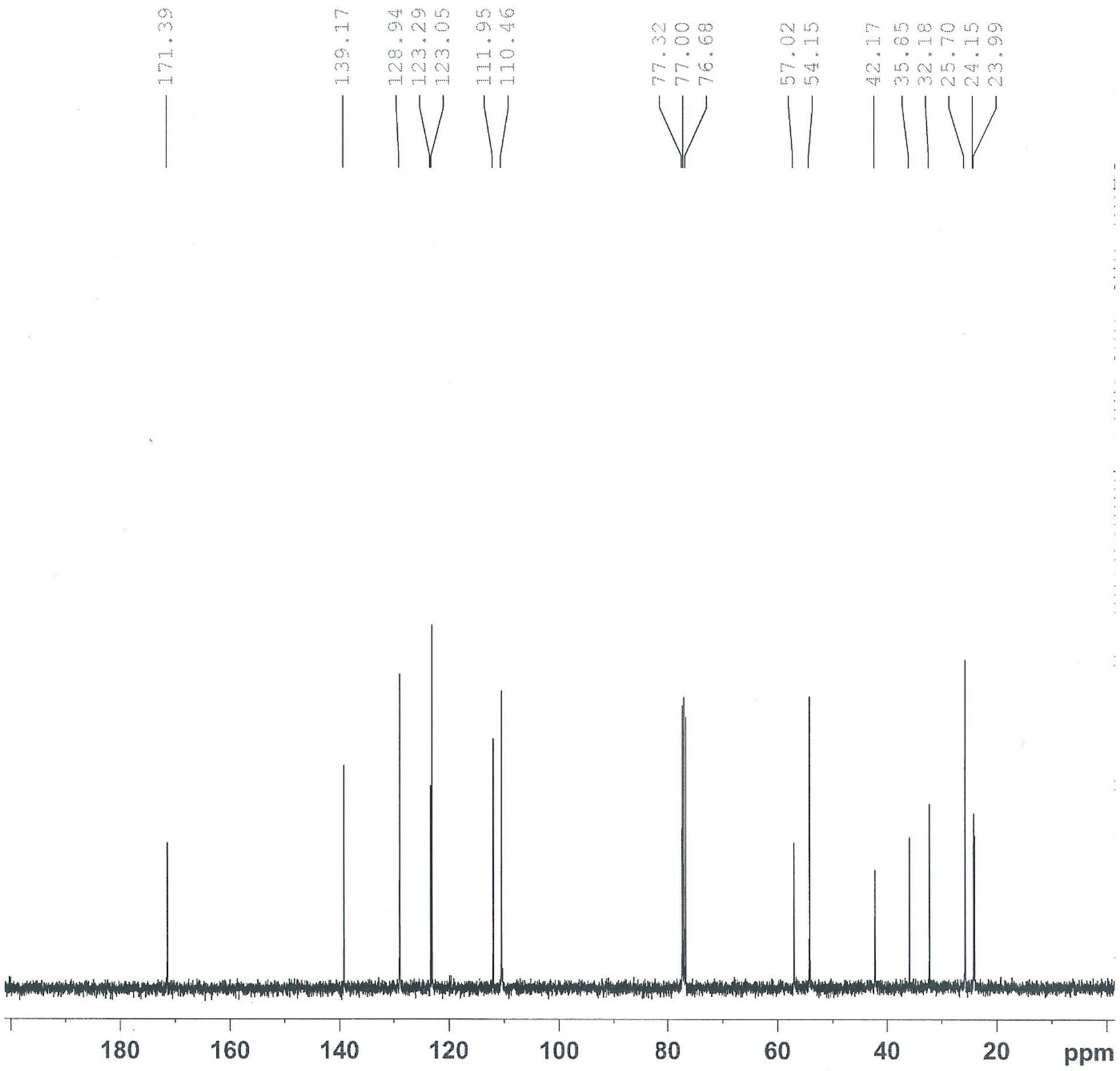

Figure S4: ^{13}C NMR Spectral Data of **7**

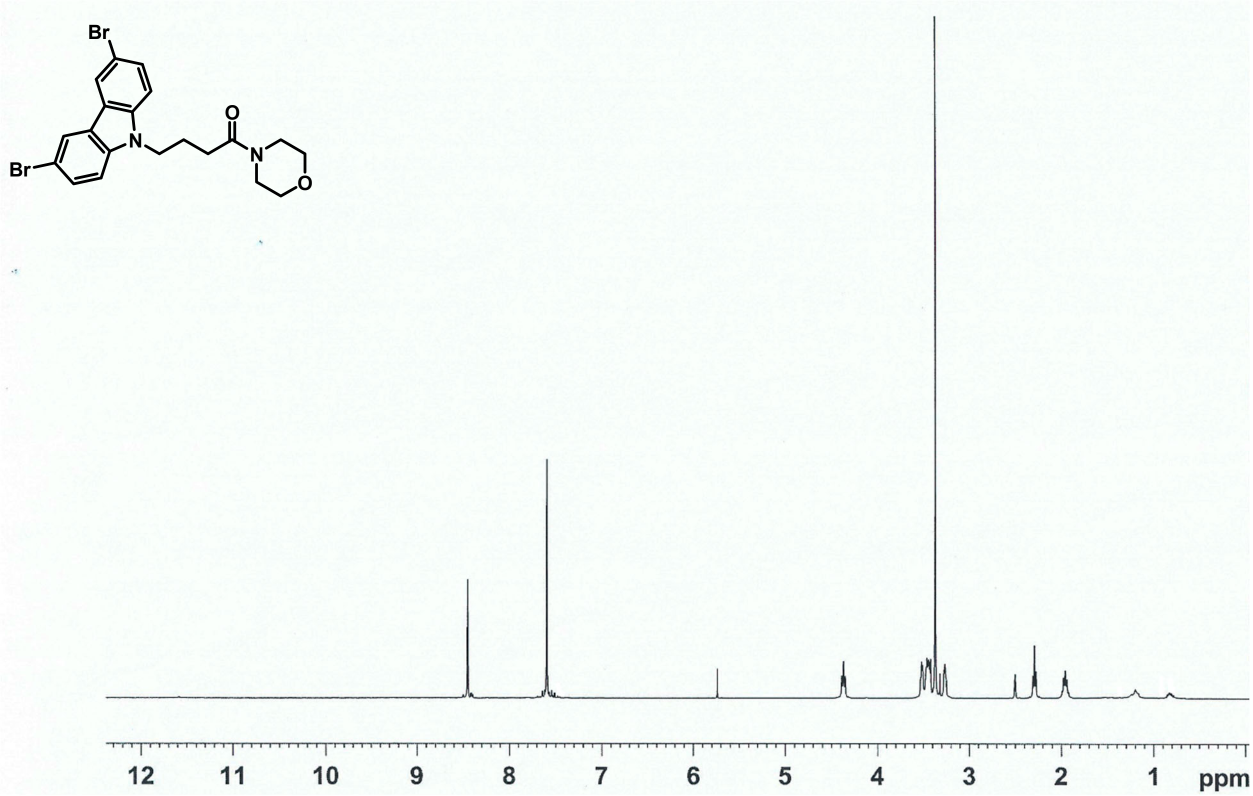

Figure S5: ^{1}H NMR Spectral Data of **12**

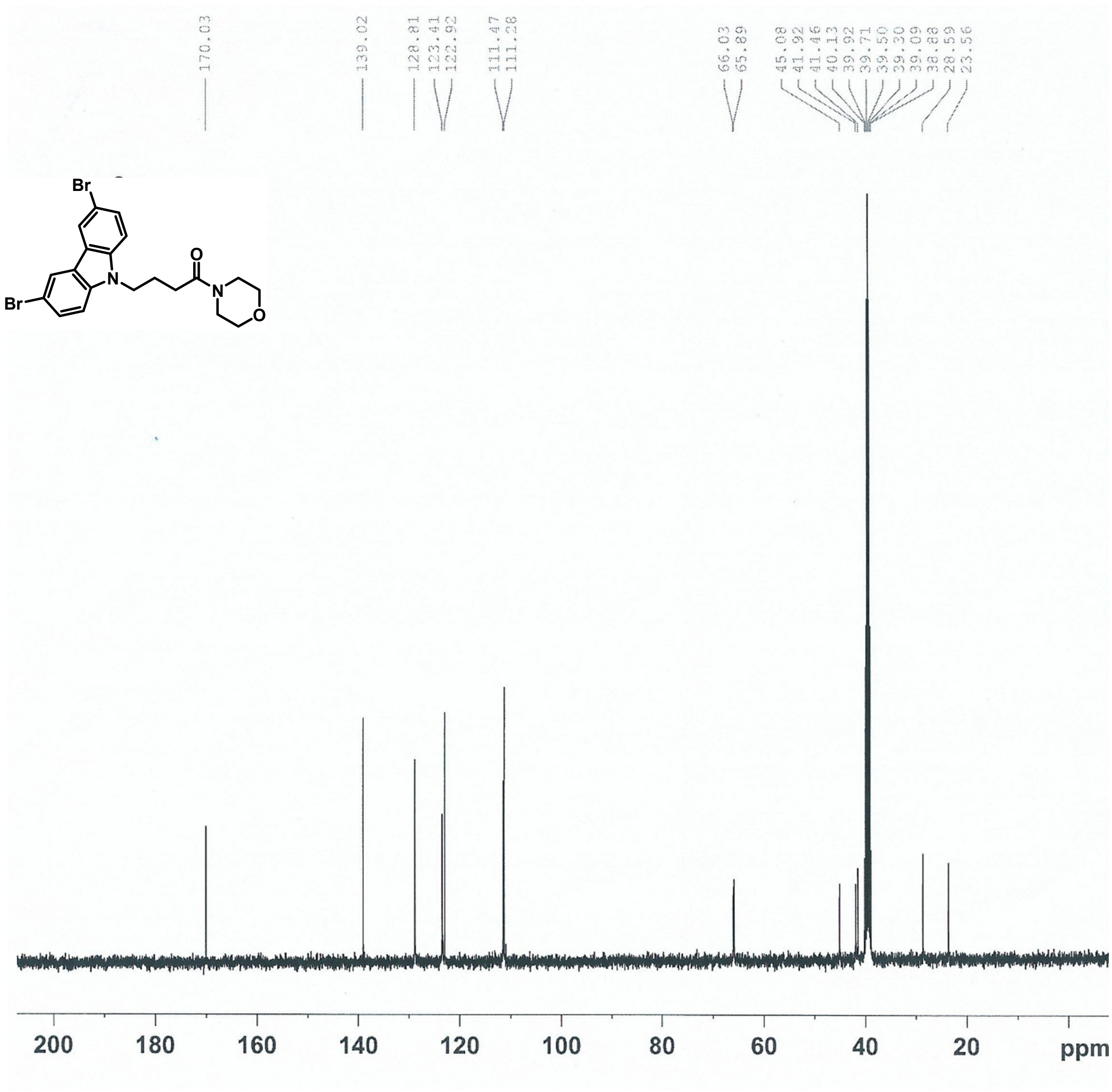

Figure S6: ^{13}C NMR Spectral Data of **12**

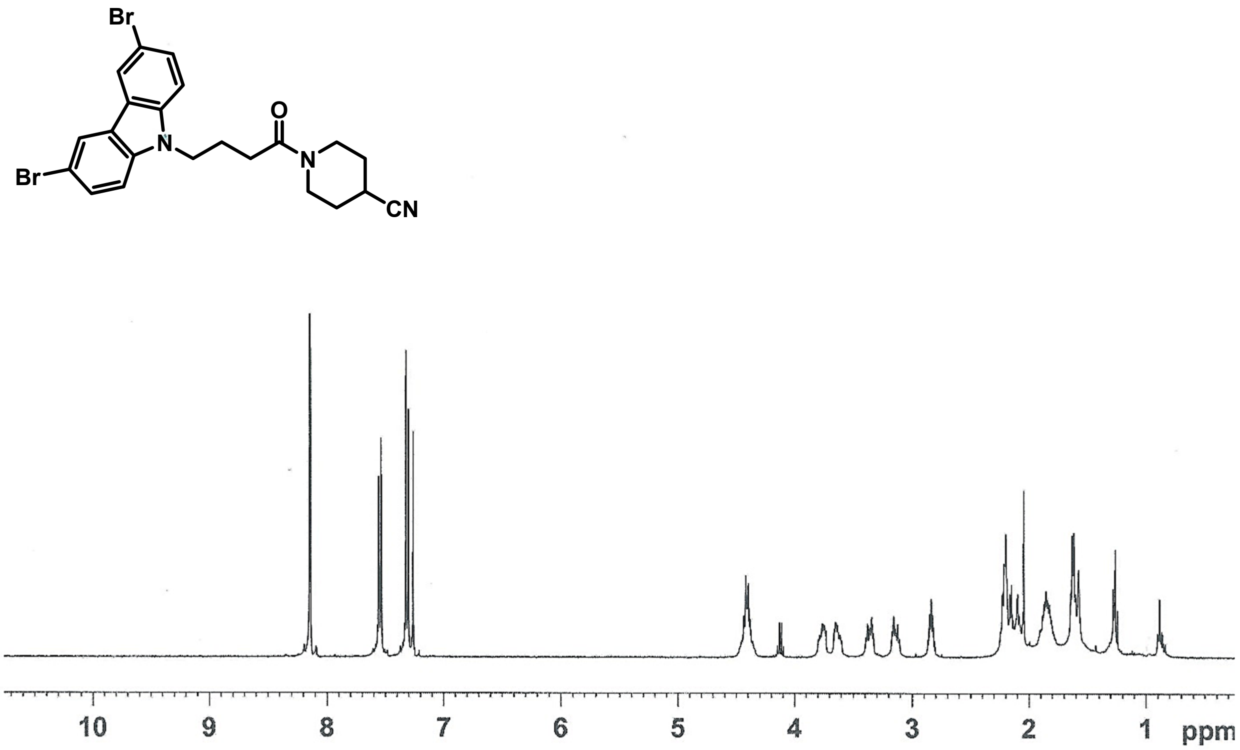

Figure S7: ^{1}H NMR Spectral Data of **14**

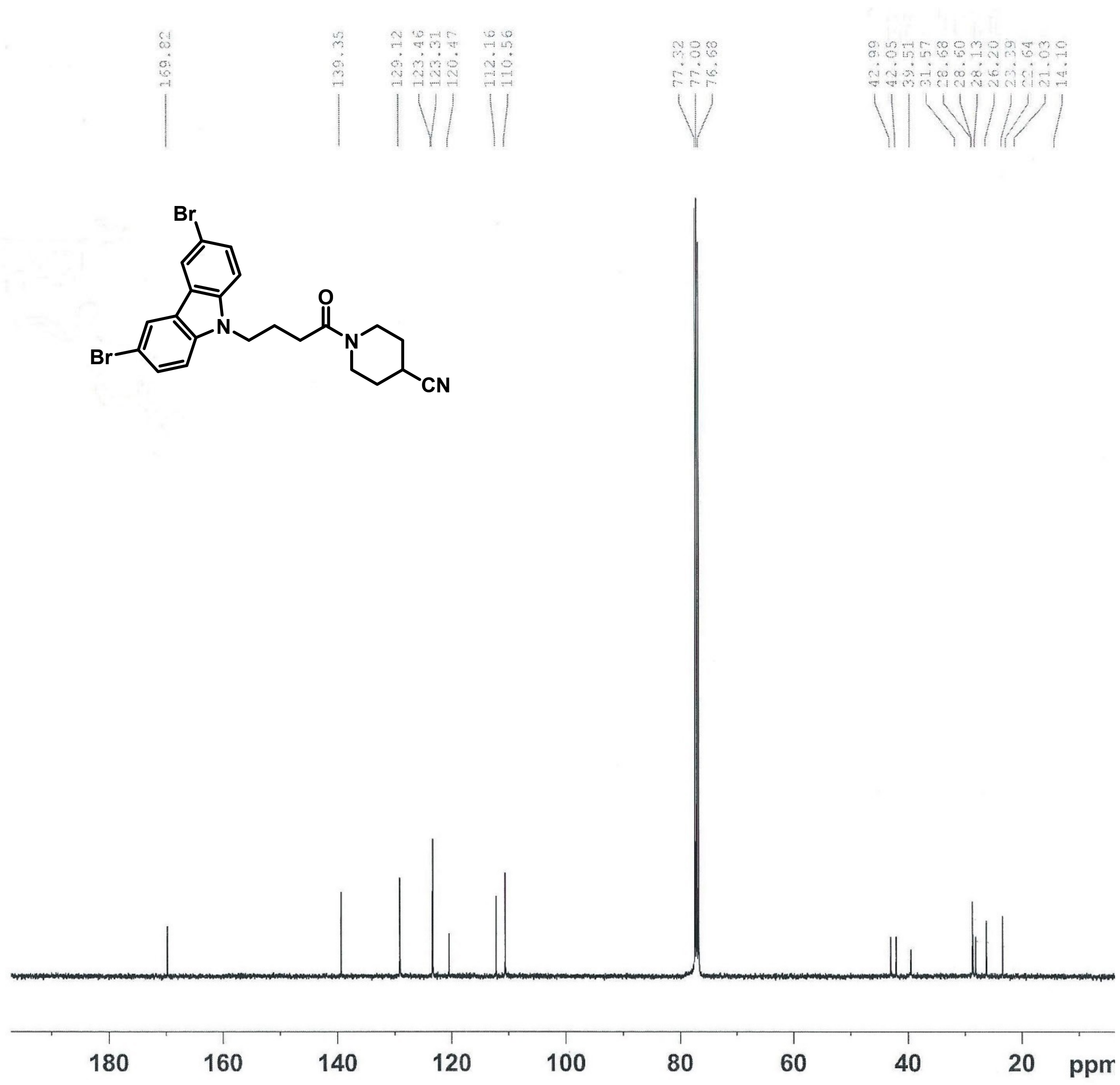

Figure S8: ^{13}C NMR Spectral Data of **14**

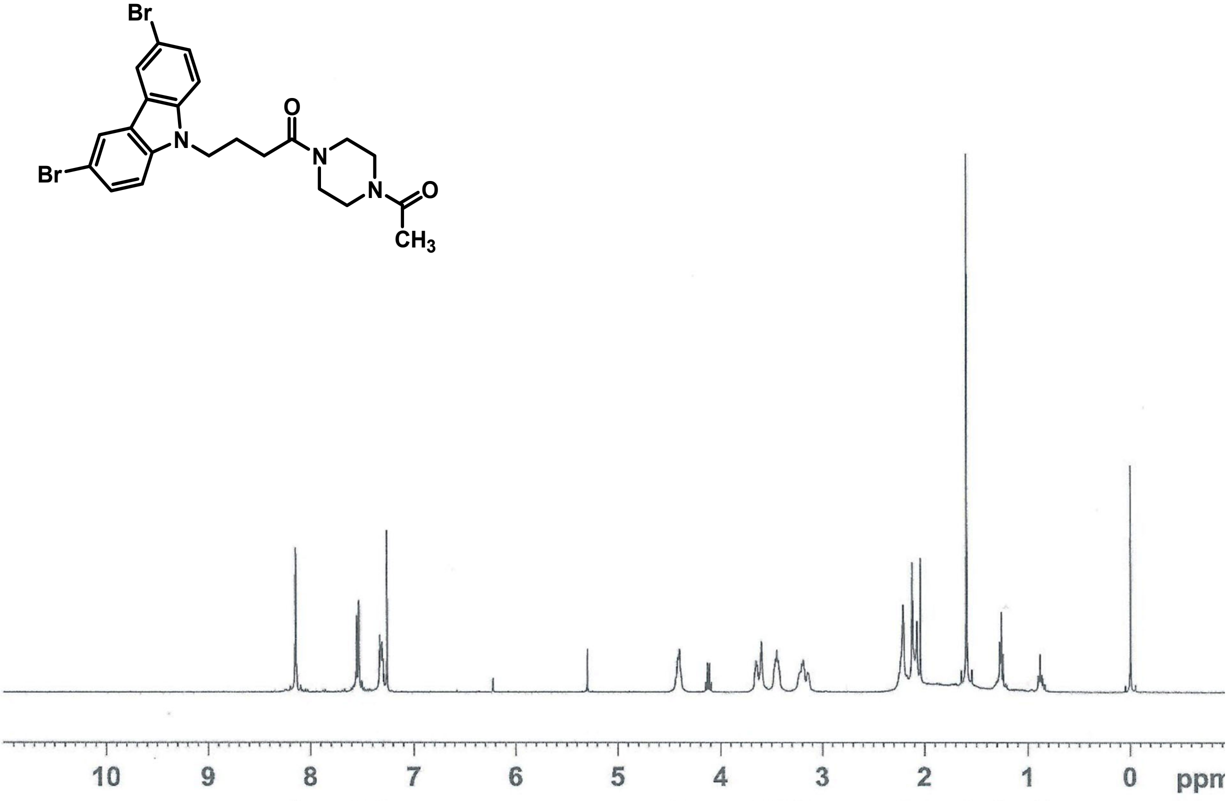

Figure S9: ^{1}H NMR Spectral Data of **16**

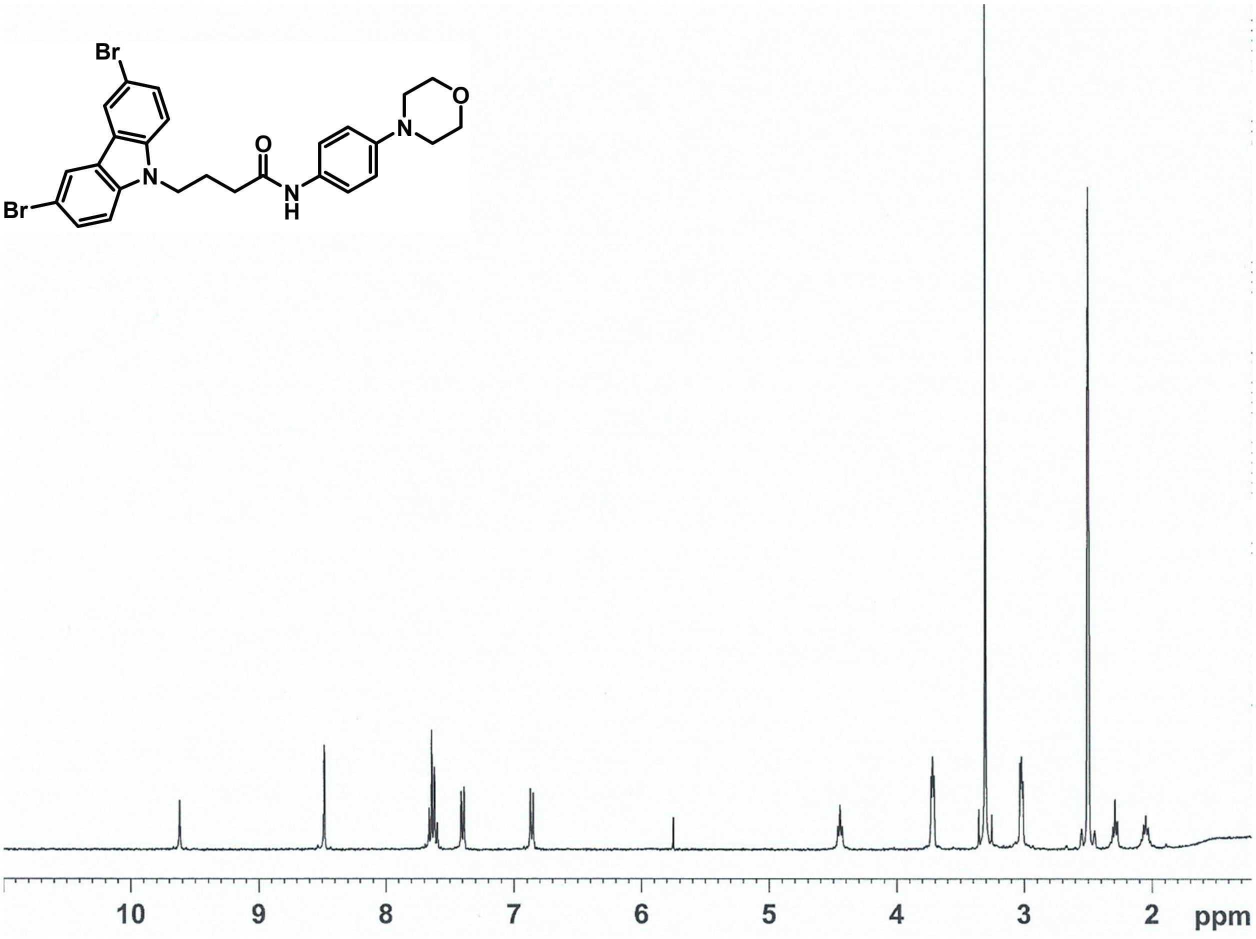

Figure S10: ^{1}H NMR Spectral Data of **22**

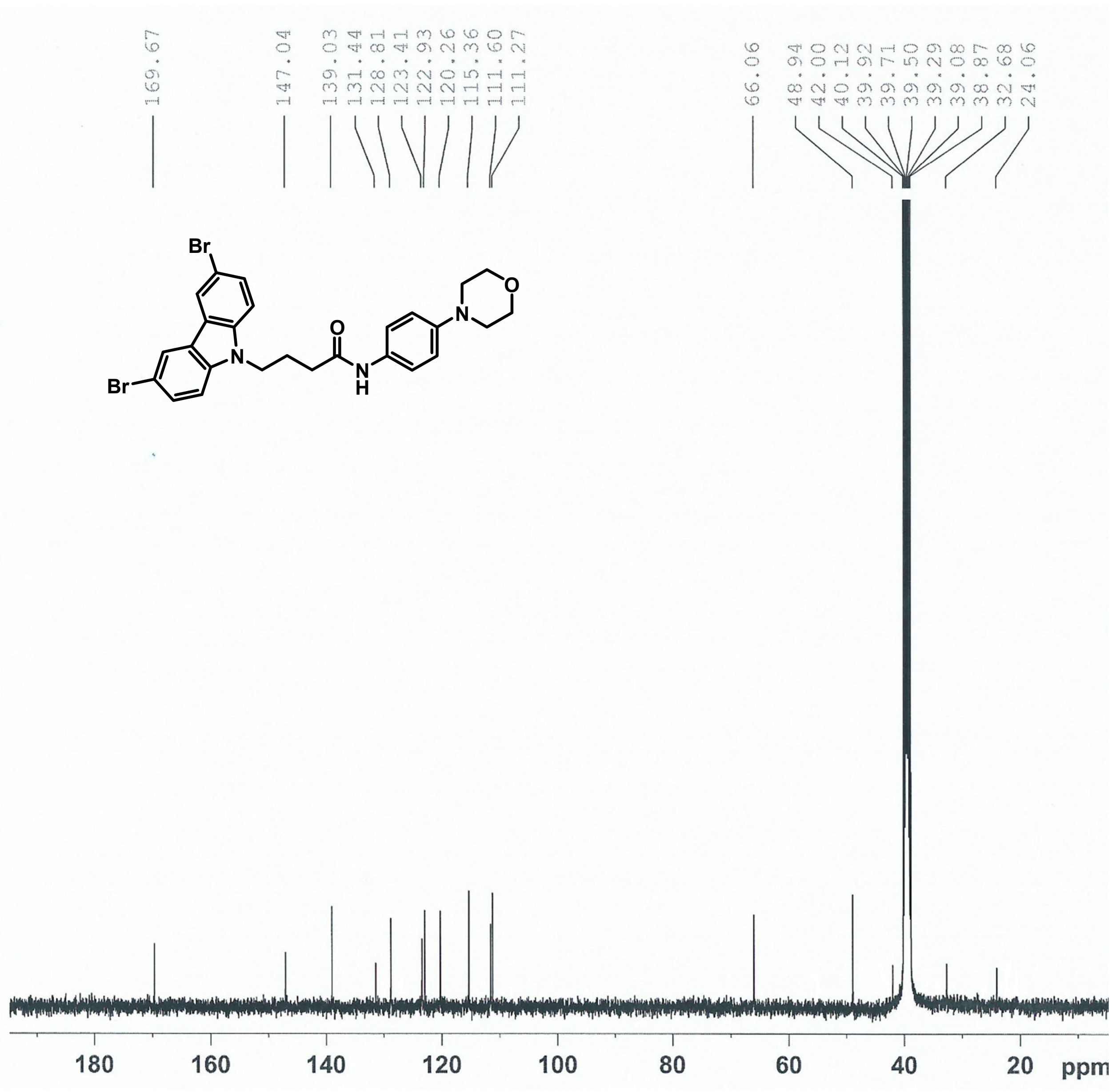

Figure S11: ^{13}C NMR Spectral Data of **22**

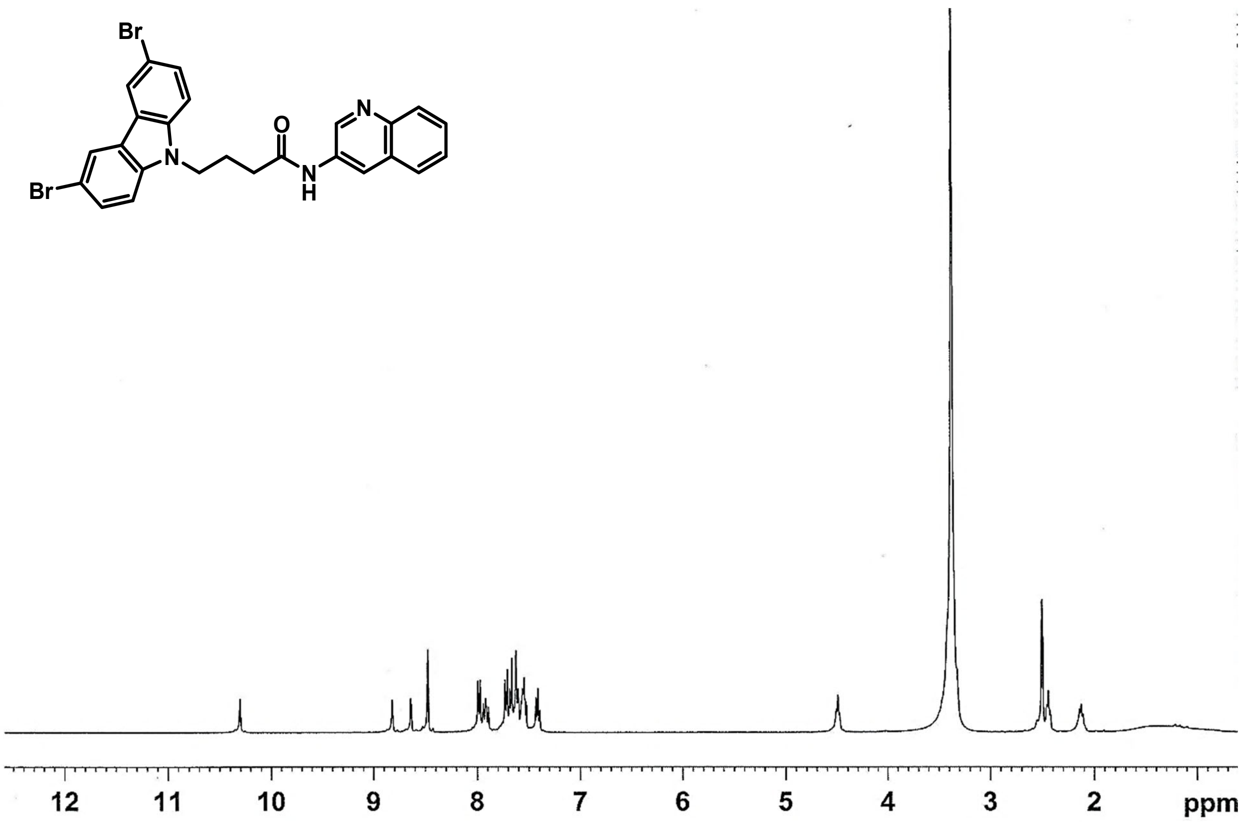

Figure S12: ^{1}H NMR Spectral Data of **24**

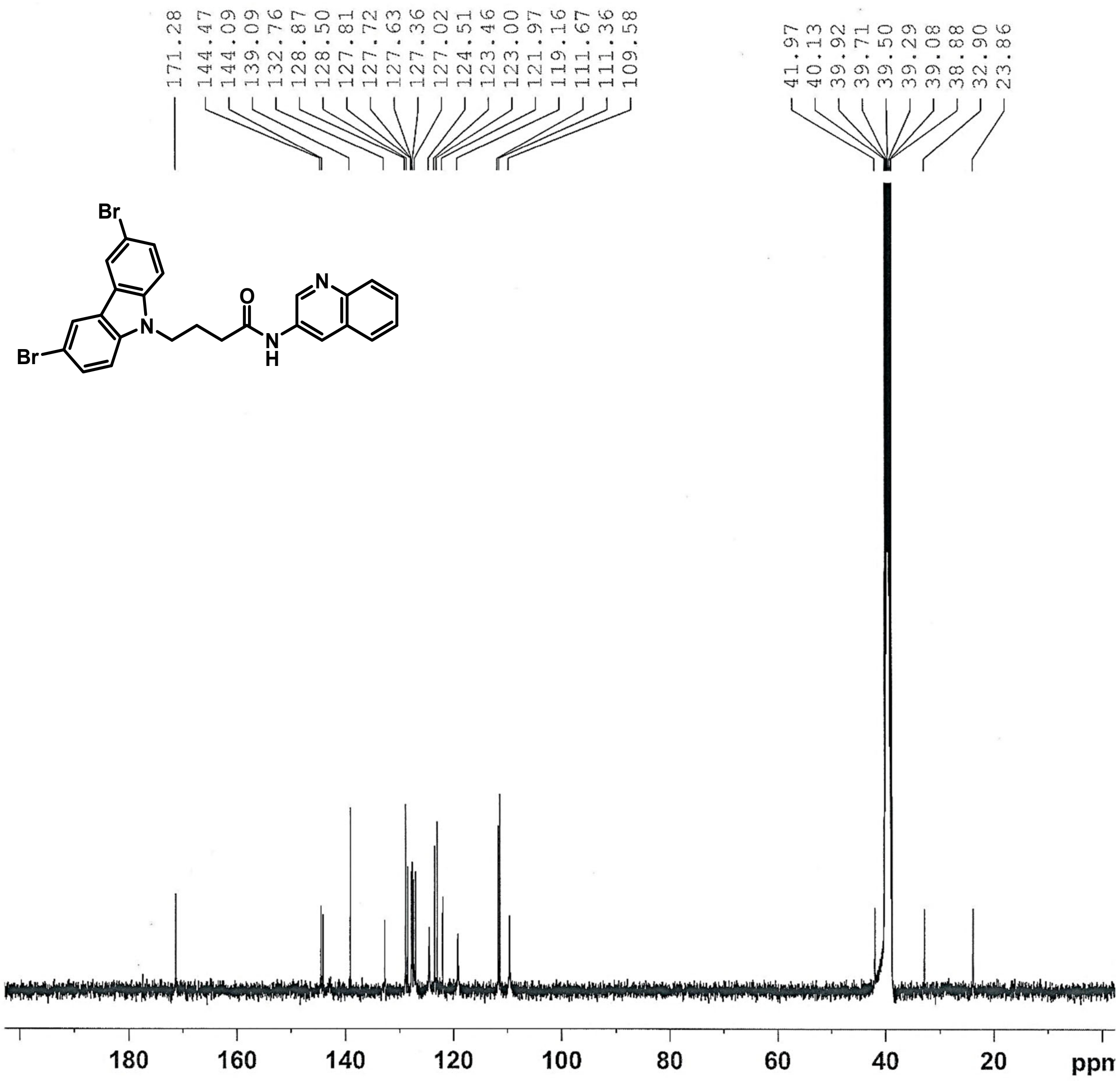

Figure S13: ^{13}C NMR Spectral Data of **24**

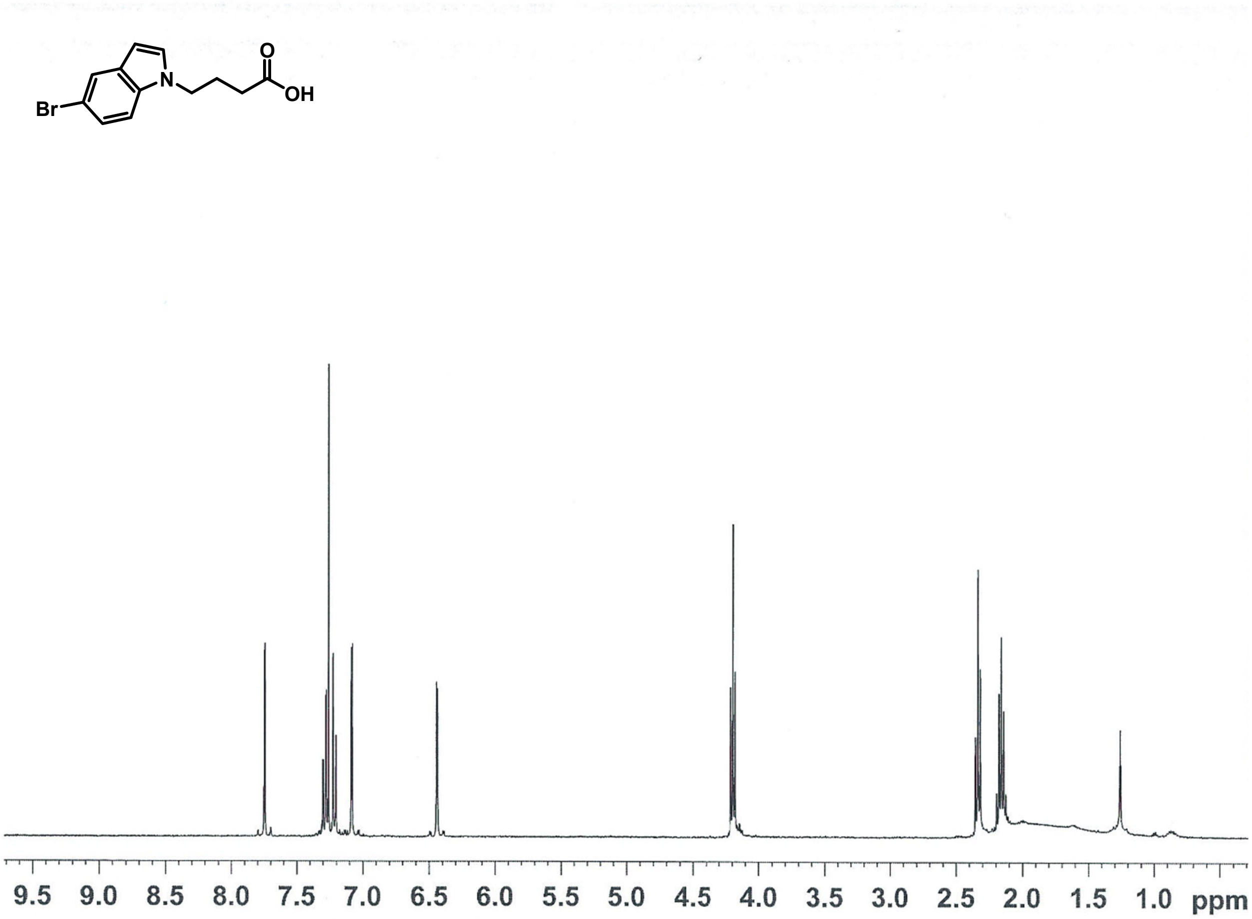

Figure S14: ^{1}H NMR Spectral Data of **29**

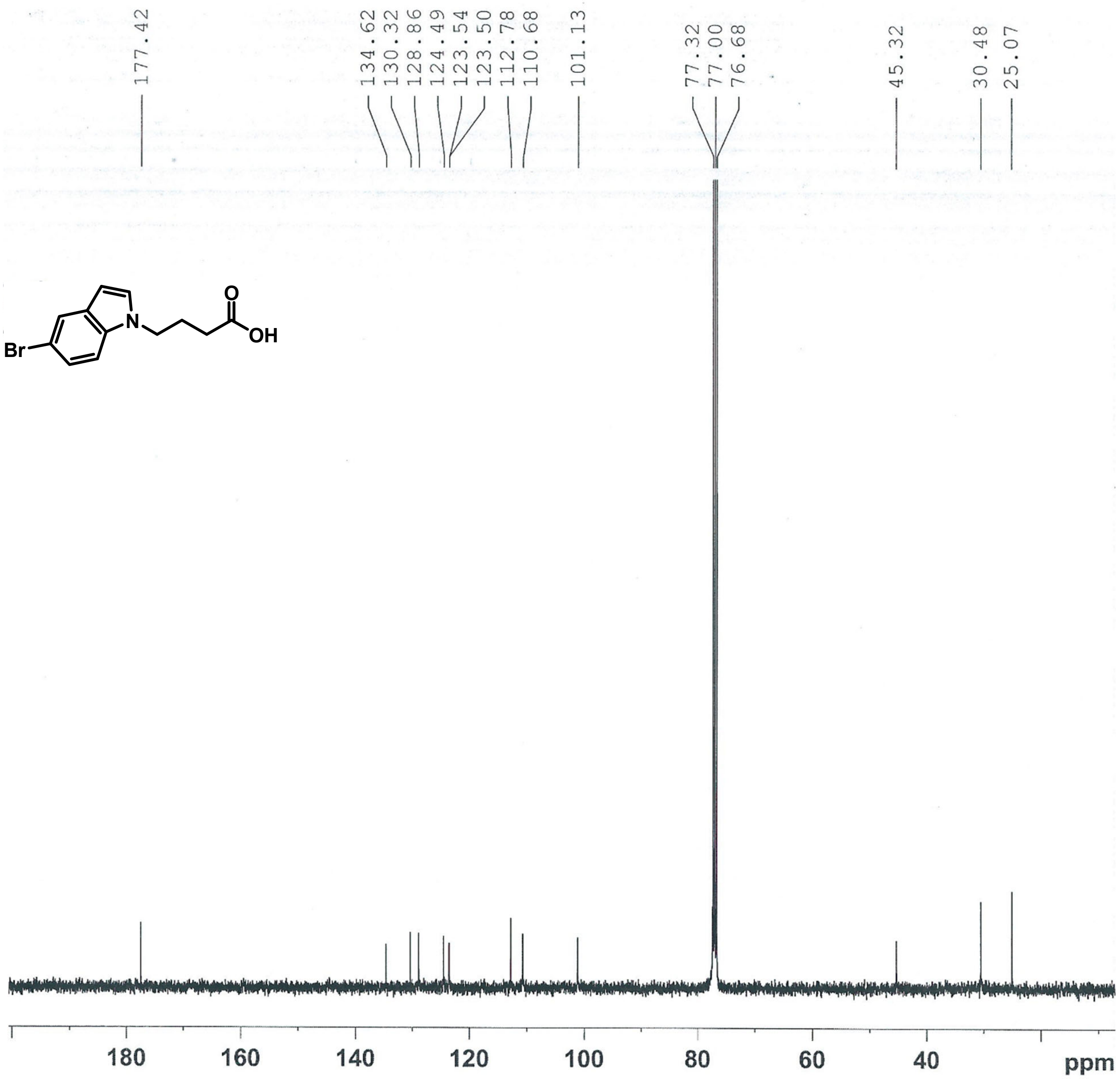

Figure S15: ^{13}C NMR Spectral Data of **29**

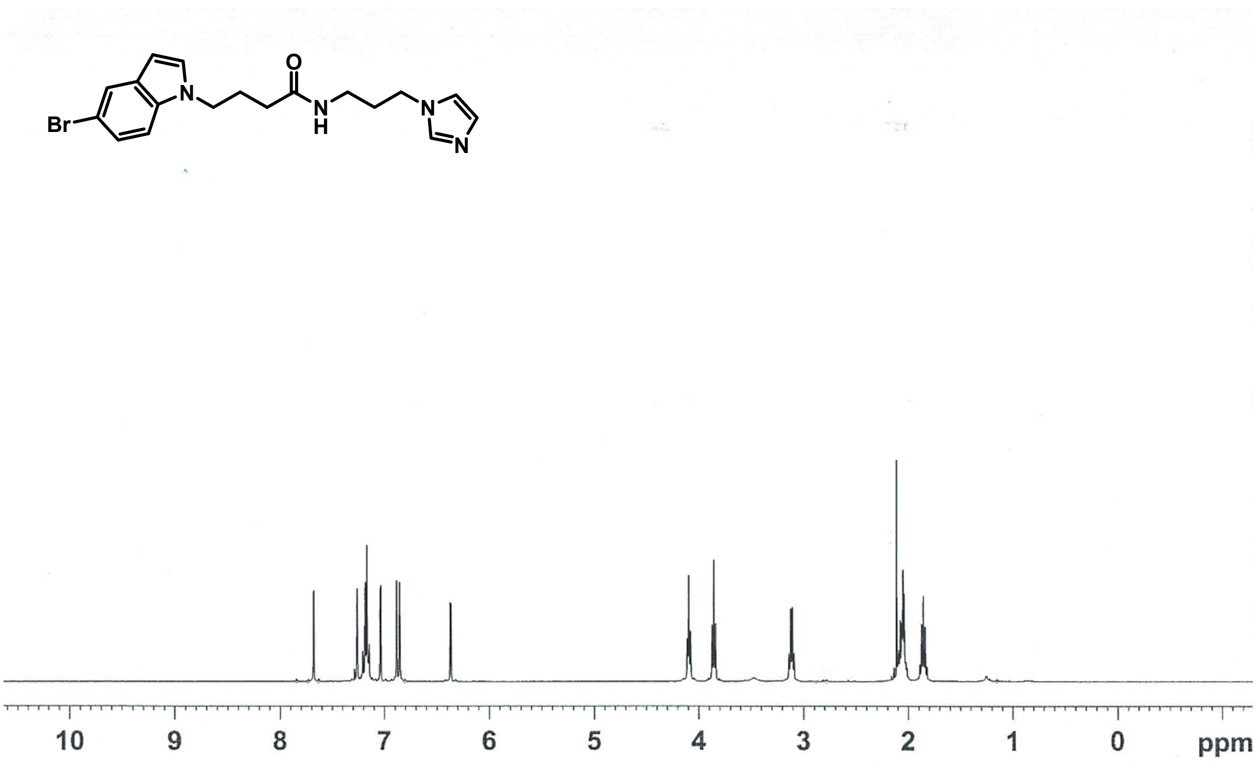

Figure S16: ^{1}H NMR Spectral Data of **33**

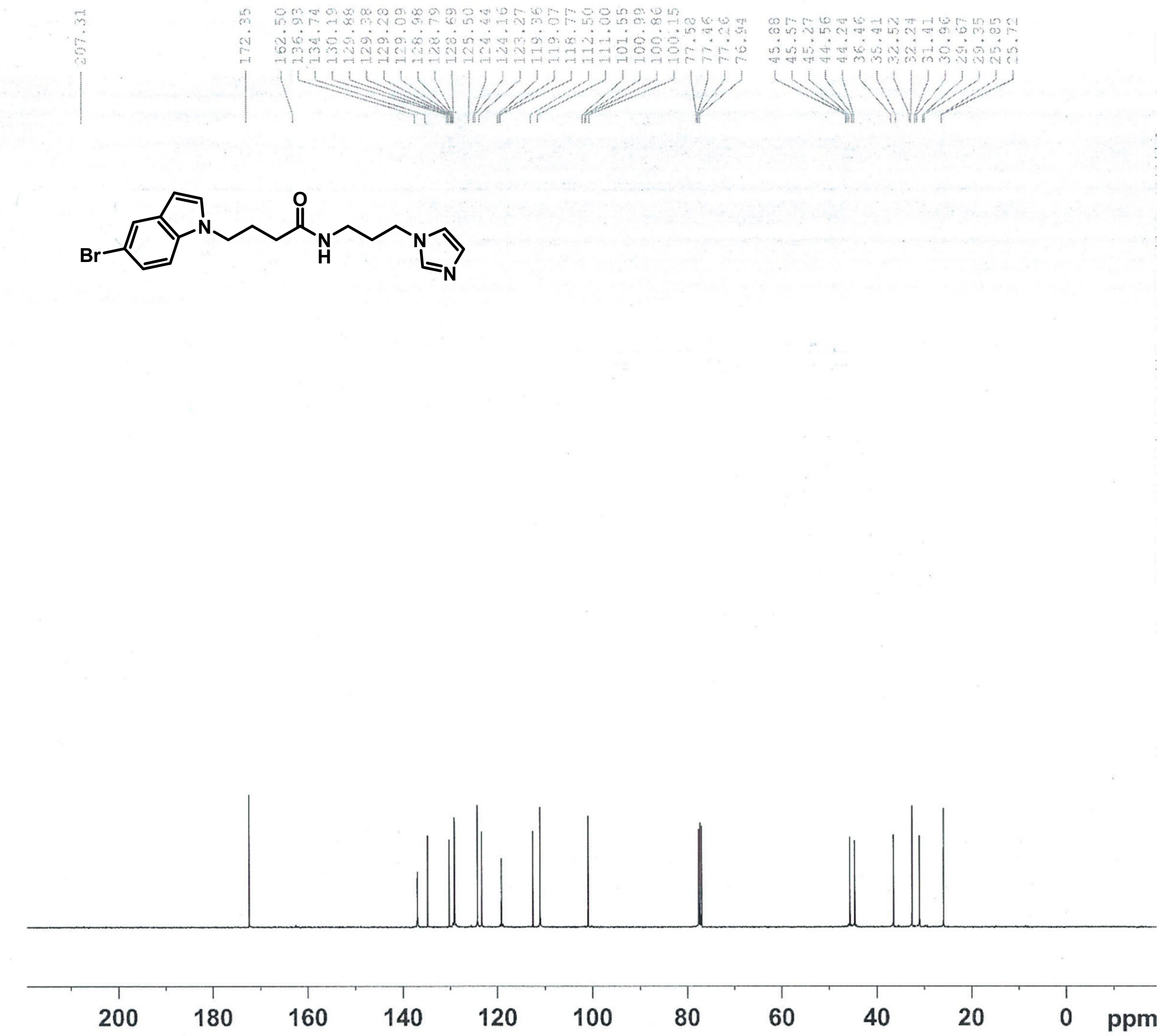

Figure S17: ^{13}C NMR Spectral Data of **33**
